# Resting Ultrasound Coronary Flow Velocity and Carotid Plaque Improve Clinical Risk Stratification in Asymptomatic Adults Undergoing Cardiovascular Screening

**DOI:** 10.3390/diagnostics16182903

**Published:** 2026-09-09

**Authors:** Nicola Gaibazzi, Davide Donelli, Pietro Renda, Marco Barbierato, Giovanni Di Salvo, Nino Carerj, Francesca Casadei, Antonella Moreo, Fausto Rigo

**Affiliations:** 1 Cardiology Department, Parma University Hospital, 43126 Parma, Italy; 2 Cardiology Unit, Ercole Franchini Hospital, AUSL–IRCCS, 42027 Reggio Emilia, Italy; davide.donelli@ausl.re.it; 3 Fondazione Villa Salus Hospital, 30174 Venice, Italy; prenda@sirm.org; 4 Cardiology Department, Ospedale dell’Angelo, 30174 Venice, Italy; marco.barbierato@aulss3.ve.it; 5 Experimental Cardiology, University of Padua, 35127 Padua, Italy; giovanni.disalvo@unipd.it; 6 Department of Clinical and Experimental Medicine, University of Messina, 98122 Messina, Italy; scipione.carerj@unime.it; 7 S.S. Imaging Cardiaco Multimodale, S.C. Cardiologia 4 Diagnostica-Riabilitativa, Niguarda Hospital, 20162 Milan, Italy; francesca.casadei@ospedaleniguarda.it (F.C.); antonella.moreo@ospedaleniguarda.it (A.M.); 8 Cardiology Division of Cardiology Center of Medicine, House of Health, 30174 Venice, Italy; faustorigo57@gmail.com

**Keywords:** echocardiography, coronary Doppler, carotid plaque, risk stratification, primary prevention, global longitudinal strain

## Abstract

**Background/Objectives**: Conventional cardiovascular risk scores may provide only modest discrimination in asymptomatic individuals undergoing screening. Ultrasound markers, including resting left anterior descending coronary artery peak diastolic flow velocity (V-LAD), carotid plaque, and global longitudinal strain (GLS), may detect subclinical abnormalities not captured by standard clinical assessment. **Methods**: In this prospective single-centre screening study, 1100 consecutive asymptomatic adults underwent clinical evaluation, laboratory testing, transthoracic echocardiography with coronary Doppler and GLS analysis, carotid ultrasound, and treadmill exercise electrocardiography. The primary analysis used Cox regression for time to the first composite event of adjudicated new-onset angina, acute coronary syndrome, heart failure hospitalization, atrial fibrillation, or death. Hierarchical models sequentially added continuous V-LAD, carotid plaque, and GLS to an internal clinical reference model. **Results**: Among 1094 evaluable participants, 79 primary endpoints occurred over a median follow-up of 25 months. Harrell C-index was 0.653 for the clinical model and increased to 0.824 after addition of V-LAD. The combined V-LAD-plus-plaque model had a C-index of 0.816, V-LAD was independently associated with outcome (HR 1.32 per + 10 cm/s (95% CI 1.23–1.43), *p* < 0.001) and carotid plaque remained strongly associated (HR 4.19 (95% CI 2.37–7.41), *p* < 0.001). Adding plaque beyond V-LAD significantly improved model fit (likelihood ratio *p* < 0.001) but did not further improve discrimination. GLS added no incremental prognostic information. **Conclusions**: V-LAD provided the major incremental gain in discrimination beyond conventional clinical variables, while carotid plaque contributed independent prognostic information and improved overall model fit without a consistent further gain in discrimination beyond V-LAD alone. GLS provided limited incremental value.

## 1. Introduction

Cardiovascular disease remains the leading cause of death in industrialized countries, and effective primary prevention relies on the accurate identification of individuals at increased risk before the occurrence of a first clinical event [1]. Established clinical risk scores such as SCORE2 in Europe and PCE/PREVENT in the United States provide valuable population-level estimates; however, their discriminative performance at the individual level remains suboptimal, with C-statistics generally ranging from 0.67 to 0.81 [2,3].

Consequently, a substantial proportion of individuals are either under- or overclassified with respect to their true cardiovascular risk. Because these models are primarily based on conventional clinical variables, they fail to capture subclinical structural and functional abnormalities that could refine individual risk stratification. In this context, although radiological methods (coronary calcium score) hold promise, fully non-invasive ultrasound imaging is particularly appealing [4]. For instance, carotid ultrasound can detect clinically silent atherosclerosis and has been shown to improve both risk discrimination and reclassification compared with SCORE2 [5,6].

Transthoracic echocardiography offers the opportunity to complement vascular imaging by providing both conventional cardiac parameters and advanced functional markers; among these, global longitudinal strain (GLS) and resting Doppler coronary flow velocity in the left anterior descending coronary artery (V-LAD) have been associated with coronary artery disease and may convey prognostic information [7,8,9].

The potential value of a combined cardiac and carotid ultrasound approach for risk discrimination and reclassification in a prospective screening setting remains insufficiently explored. If such an approach demonstrates incremental value beyond conventional clinical predictors, it could support a more individualized ultrasound-based strategy for primary cardiovascular prevention.

The PENSIAMOCI-PRIMA study [10] is a prospective, single-center cohort of asymptomatic individuals undergoing cardiovascular screening, designed to evaluate whether cardiac and carotid ultrasound markers improve risk stratification beyond conventional clinical risk scores and biomarkers. In the present analysis, we specifically aimed to assess whether V-LAD, carotid plaque and GLS provide incremental prognostic information beyond a parsimonious internal clinical reference model, with SCORE2 used as a contextual comparator.

## 2. Methods

### 2.1. Study Design and Population

This is an interim analysis of the PENSIAMOCI-PRIMA study, aiming at combined cardiac outcome, using mid-term follow-up data. The study is a prospective, single-centre, voluntary cardiovascular screening program designed to test the research hypothesis that preclinical detection of coronary or systemic atherosclerosis using resting ultrasound imaging improves risk stratification of asymptomatic individuals beyond standard clinical assessment.

The study is conducted in the greater Venice area and is funded by an unrestricted grant from a private charitable foundation (Ifis Bank Foundation). For the present analysis, we included the first 1100 consecutive participants enrolled between study initiation in May 2023 and February 2025. Screening was offered free of charge through public advertisement to self-referred asymptomatic adults aged 45 to 69 years who agreed to participate and provided written informed consent. The study was conducted in accordance with the Declaration of Helsinki. All participants underwent a structured, same-day screening visit including clinical assessment, resting 12-lead electrocardiogram, fasting blood sampling, comprehensive transthoracic echocardiography, carotid ultrasound, and treadmill exercise electrocardiography. All participants were free of angina and anginal equivalents at enrolment; individuals with suspicious symptoms were not eligible for the asymptomatic screening cohort. The objective of the present analysis was to evaluate whether preselected ultrasound-based markers improve short-term prediction of a composite cardiovascular endpoint beyond a parsimonious internal clinical reference model.

### 2.2. Baseline Assessment and Data Collection

Baseline assessment was standardized and performed on the same day for all participants. Clinical data collection included medical history, documentation of cardiovascular risk factors, physical examination, resting electrocardiography, fasting blood testing, comprehensive transthoracic echocardiography, and bilateral carotid duplex ultrasound. Recorded clinical variables included family history of coronary artery disease, hypertension, hypercholesterolemia, diabetes mellitus, current or former smoking status, sedentary lifestyle, and extracardiac comorbidities. Physical examination included measurement of abdominal circumference, body surface area, and body mass index, as well as systolic and diastolic blood pressure measured in both arms and cardiac auscultation.

Electrocardiographic variables included heart rate, PR interval, QRS duration, and corrected QT interval. Laboratory testing included glucose, creatinine, uric acid, C-reactive protein, NT-proBNP, triglycerides, total cholesterol, HDL cholesterol, calculated LDL cholesterol, directly measured LDL cholesterol, thyroid-stimulating hormone, and complete blood cell counts.

SCORE2 for moderate-risk regions was calculated using available clinical variables and was used solely as a contextual comparator in the present analysis.

### 2.3. Echocardiographic and Carotid Ultrasound Acquisition

All participants underwent comprehensive transthoracic echocardiography using a GE Vivid E95 ultrasound platform (GE Vingmed Ultrasound AS, Horten, Norway) equipped with an M5S-D phased-array cardiac transducer for transthoracic echocardiography and coronary Doppler assessment, and a GE 9L-D high-frequency linear transducer for carotid ultrasound. Standard parasternal long-axis and short-axis views and apical 2-, 3-, and 4-chamber views were acquired with the patient in the left lateral decubitus position by experienced echocardiographers. Conventional measurements, including chamber dimensions, left ventricular volumes, left ventricular ejection fraction, wall thickness, left ventricular mass, relative wall thickness, and left atrial volume, were obtained in accordance with current ASE/EACVI recommendations [11]. Global longitudinal strain (GLS) was assessed using speckle-tracking echocardiography from apical 2-, 3-, and 4-chamber views, with a frame rate of 60–90 frames/s. Offline analysis was performed using dedicated software (EchoPAC, version 204, GE Vingmed Ultrasound AS, Horten, Norway); endocardial borders were manually adjusted when necessary, and GLS was calculated as the average peak systolic strain across all left ventricular segments.

Resting coronary flow in the left anterior descending artery (LAD) was assessed by transthoracic Doppler echocardiography using the same cardiac transducer. Coronary flow was identified in modified parasternal short-axis or low parasternal (or apical) long-axis view, along the anterior interventricular groove, and focused on the interventricular septum. Color Doppler was optimized with high myocardial wall filtering and a low Nyquist limit (20–30 cm/s) and increased gain to detect coronary flow, followed by pulsed-wave Doppler to measure peak diastolic flow velocity over at least three consecutive cardiac cycles. In the present analysis, the main coronary Doppler variable was the resting peak diastolic flow velocity measured at the second segment of the LAD. Secondary threshold-based analyses were performed using a peak diastolic velocity of 45 cm/s as the upper limit of normal for the mid-LAD, consistent with published normative data [12].

V-LAD acquisition was attempted in all participants. An analyzable V-LAD value was available in at least 1091 of 1094 evaluable participants (99.7%). A dedicated inter- or intra-observer reproducibility substudy was not performed in this cohort. Prior studies from experienced laboratories have reported high reproducibility for transthoracic coronary Doppler measurements [13].

Bilateral carotid ultrasound was performed using a high-resolution linear transducer according to a standardized protocol. The common carotid artery, carotid bifurcation, internal carotid artery, and external carotid artery were examined bilaterally. Carotid plaque was defined as a focal structure encroaching into the arterial lumen by at least 0.5 mm or 50% of the surrounding intima-media thickness, or with a thickness > 1.5 mm. For the purposes of the present analysis, the presence of any carotid plaque was defined as plaque detected in either the common or internal carotid artery.

### 2.4. Endpoint Definition

The primary endpoint was time to the first occurrence of the prespecified composite of adjudicated new-onset angina, acute coronary syndrome, hospitalization for heart failure, atrial fibrillation, or death. All 67 angina events represented symptoms developing after enrolment in participants who were free of angina or anginal equivalents at baseline. These events led to evaluation in an emergency department or cardiology ward and were clinically classified according to the Canadian Cardiovascular Society grading system. For endpoint adjudication, new-onset angina additionally required objective evidence of ischemia on exercise electrocardiography or stress echocardiography and/or coronary CT angiography demonstrating at least one coronary stenosis ≥ 50%. Revascularization procedures (PCI or CABG) were not components of the endpoint. For participants experiencing an endpoint, follow-up time was calculated from enrolment to the date of the first qualifying event or symptom onset; for participants remaining event-free, follow-up extended from enrolment to the last available clinical follow-up.

Downstream coronary testing was clinically selective. CCTA was generally not performed when V-LAD was normal and the treadmill exercise test was negative, except in rare participant-requested examinations. CCTA was generally pursued when V-LAD was ≥45 cm/s together with a positive or equivocal treadmill test, or when V-LAD exceeded 50 cm/s irrespective of treadmill findings. The cardiologists interpreting stress tests and the radiologists interpreting CCTA were not informed of the screening reason prompting referral. Because V-LAD itself could contribute to referral for CCTA, referral and partial-verification bias cannot be completely excluded.

A prespecified sensitivity analysis used a more restrictive endpoint excluding angina. In addition, to address the inclusion of non-coronary cardiac events in the prespecified composite, a coronary-specific sensitivity analysis restricted to angina plus acute coronary syndrome was performed.

### 2.5. Candidate Variables and Model Building

The main ultrasound candidate variables were V-LAD, GLS, and the presence of any carotid plaque. In the primary analyses, both V-LAD and GLS were modelled as continuous variables in the extended imaging models. Secondary, clinically oriented analyses explored binary and three-category classifications based on empirically derived thresholds; however, these categorizations were not used in the main multivariable models. To provide an appropriate clinical benchmark, a parsimonious internal reference model was derived from a prespecified set aligned with SCORE2 domains: age, sex, smoking status, systolic blood pressure, total cholesterol, and HDL cholesterol. All variables were initially included in a multivariable logistic model; HDL cholesterol showed no meaningful contribution and was removed to preserve parsimony. The final clinical model (M0) therefore included age, male sex, smoking, systolic blood pressure, and total cholesterol.

Imaging models were constructed hierarchically: M1 included M0 plus V-LAD; M2 included M0 plus carotid plaque; M3 included M0 plus V-LAD and carotid plaque; and M4 included M3 plus GLS. Additional nested comparisons assessed the incremental value of carotid plaque beyond V-LAD and of GLS beyond the combined V-LAD-plus-plaque model.

SCORE2 was calculated from available variables and analyzed in its ordinal categorical form to reflect routine clinical use. As it was developed for a different endpoint and time horizon, it was used only as a contextual comparator rather than as the primary reference model.

### 2.6. Statistical Analysis

Continuous variables are presented as median and interquartile range, and categorical variables as counts and percentages. Baseline characteristics were described overall and stratified by primary endpoint status, with between-group comparisons reported in the descriptive tables.

The primary prognostic analysis used Cox proportional hazards regression with time to the first primary endpoint as the outcome. Model discrimination was summarized using Harrell’s C-index, and incremental value across nested models was evaluated with likelihood ratio tests and changes in C-index on matched rows. Bootstrap resampling (500 replicates) was used to characterize uncertainty in selected changes in C-index. The proportional hazards assumption was evaluated using Schoenfeld residuals. The global proportional hazards test was non-significant for all main models. A nominal deviation was observed for systolic blood pressure in some models; therefore, the final inferential Cox models incorporated the same systolic blood pressure × log(time/24) interaction across M0–M4, while V-LAD, carotid plaque, and GLS were modelled with constant hazard ratios. No evidence of a time-varying V-LAD effect was observed. Complementary logistic analyses are reported in the Supplementary Materials, including AUC, Brier score, bootstrap internal validation, NRI/IDI, SCORE2 contextual comparison, threshold analyses, and decision curve analysis. All models used available cases for the variables entering the specific analysis.

## 3. Results

### 3.1. Study Sample and Endpoint Distribution

The screened cohort included 1100 asymptomatic individuals. Six participants had missing adjudicated primary endpoint information and were excluded from the present analysis; therefore, the final analytic sample for the primary endpoint comprised 1094 subjects, of whom 79 (7.2%) experienced the primary composite endpoint and 1015 (92.8%) did not (Figure S1, study flowchart).

The primary endpoint was driven predominantly by new-onset angina (*n* = 67), followed by acute coronary syndrome (*n* = 6), atrial fibrillation (*n* = 5), and heart failure (*n* = 1); no deaths occurred. All angina cases developed after enrolment and were evaluated in an emergency department or cardiology ward. Coronary revascularization procedures during follow-up (PCI, *n* = 56; CABG, *n* = 11) are reported descriptively only and were not included in the endpoint. Median follow-up was 25 months (interquartile range, 22–27 months). Event time and censoring time were available for all 1094 participants with classifiable endpoint status.

### 3.2. Baseline Characteristics

Overall baseline characteristics and according to primary endpoint status are shown in Table 1. Compared with individuals without the primary endpoint, those experiencing the endpoint were slightly older (62.0 [58.5–67.0] vs. 60.0 [56.0–65.0] years, *p* = 0.035), more frequently male (63.3% vs. 49.2%, *p* = 0.0218), and had a higher prevalence of hypertension (55.7% vs. 33.6%, *p* < 0.001) and hypercholesterolemia (75.9% vs. 55.4%, *p* < 0.001). Systolic blood pressure was also higher in the endpoint group (132 [131–145] vs. 130 [120–140] mmHg, *p* < 0.001). In contrast, smoking status and family history of coronary artery disease did not differ significantly between groups.

The main imaging variables showed the most pronounced between-group differences. V-LAD was substantially higher in individuals with the primary endpoint compared with those without (56.0 [49.0–63.5] vs 34.0 [33.0–40.0] cm/s, *p* < 0.001), and the presence of any carotid plaque was markedly more frequent (70.9% vs. 28.5%, *p* < 0.001). Global longitudinal strain was less negative in the endpoint group (−19.0% [−21.0 to −18.0] vs. −21.0% [−22.0 to −19.0], *p* < 0.001). Supplementary Figure S2 graphically shows the distribution of V-LAD, GLS and carotid plaque status according to primary endpoint status.

Downstream clinically driven diagnostic testing was more frequently performed among participants who subsequently met the primary endpoint, reflecting the adjudication pathway for the angina component. CT coronary angiography was performed in 73/79 participants with the primary endpoint and in 247/1015 without the endpoint; invasive coronary angiography was performed in 9/79 and 63/1015, respectively. These differences reflect the clinically selective downstream testing pathway described above and should not be interpreted as uniform verification of coronary anatomy.

### 3.3. Internal Clinical Reference Model

The internal clinical reference model (M0) was derived from the prespecified 6-variable candidate set aligned with the SCORE2 domains. After simultaneous multivariable fitting, HDL cholesterol was excluded as the weakest predictor, and the final parsimonious model retained age, male sex, smoking, systolic blood pressure, and total cholesterol.

In the primary Cox analysis, M0 showed modest discrimination (Harrell C-index 0.653). Male sex was associated with the primary endpoint (HR 1.77; 95% CI 1.10–2.85; *p* = 0.018). Because systolic blood pressure showed evidence of non-proportional hazards, its effect was time-dependent, with an HR per 10-mmHg increase of 0.96 (95% CI 0.76–1.21) at 12 months, 1.28 (95% CI 1.10–1.48) at 24 months, and 1.51 (95% CI 1.18–1.93) at 36 months.

### 3.4. Incremental Value of Imaging Variables

**Figure 1:**
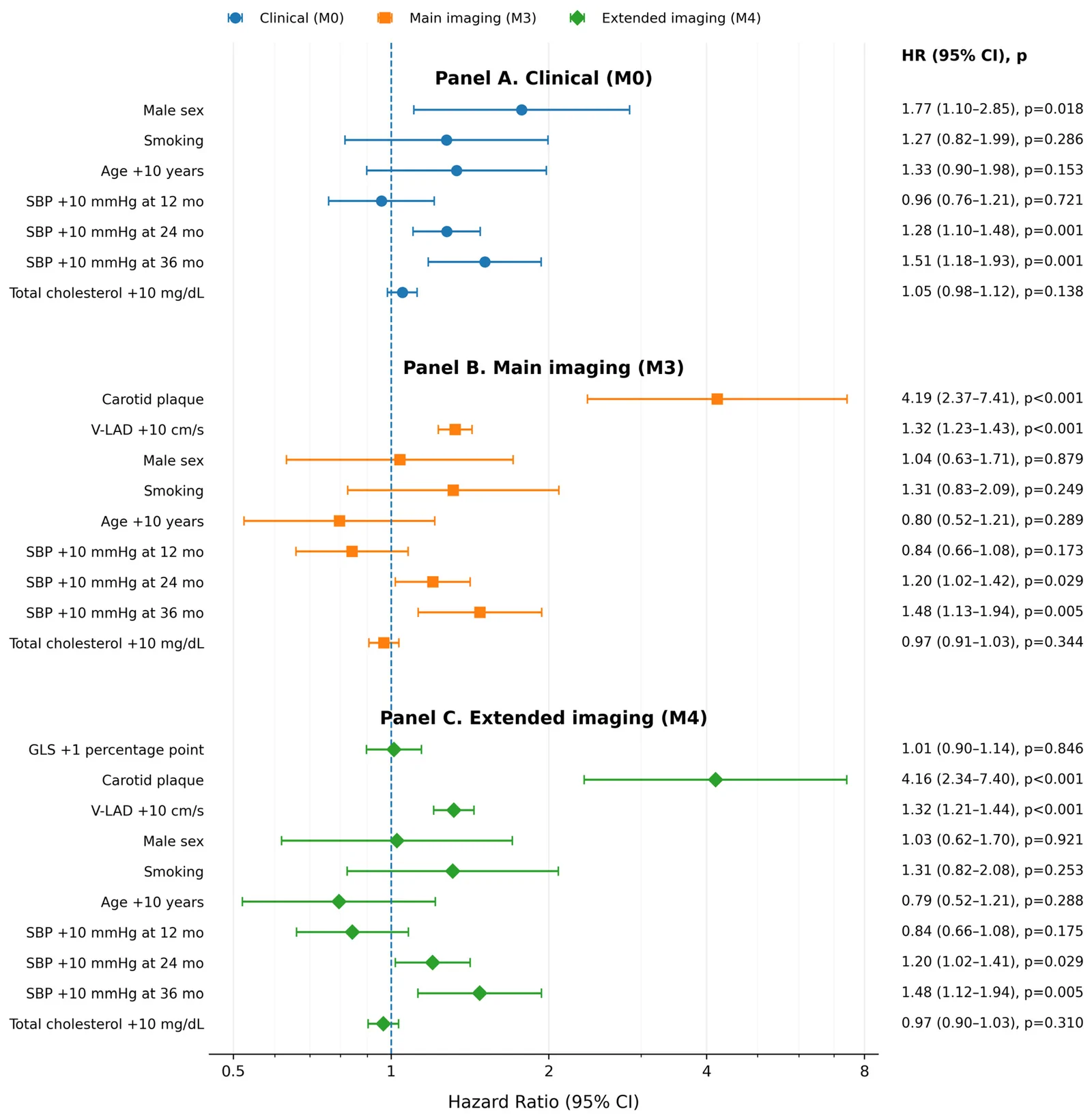
Forest plot of the hazard ratios of the internal clinical reference model (M0), main imaging model (M3) and extended imaging model (M4). SBP hazard ratios are reported at 12, 24, and 36 months to illustrate its time-varying effect.

Model performance across the sequential Cox models is summarized in Table 2, while Figure 1 shows the forest plots of the multivariable coefficients for the internal clinical model and the two most relevant imaging models with added ultrasound imaging.

In the primary time-to-event analysis, adding continuous V-LAD to the clinical model (M1 vs. M0) markedly improved discrimination, with the C-index increasing from 0.653 to 0.824. The combined imaging model (M3), integrating the clinical model with continuous V-LAD and any carotid plaque, had a C-index of 0.816 and significantly improved model fit compared with M0 (likelihood ratio *p* < 0.001). In multivariable Cox analysis, both V-LAD and carotid plaque remained independently associated with the primary endpoint. The HR was 1.028 per 1 cm/s increase in V-LAD (95% CI, 1.021–1.036; *p* < 0.001), corresponding to approximately 1.32 per 10 cm/s, and 4.19 for any carotid plaque (95% CI, 2.37–7.41; *p* < 0.001).

In a targeted comparison restricted to matched rows, adding carotid plaque beyond the model containing clinical variables and V-LAD (M3 vs. M1) significantly improved model fit (likelihood ratio *p* < 0.001), although the C-index did not increase (0.824 vs. 0.816). Consistently, bootstrap analysis showed a mean change in C-index from M1 to M3 of −0.0038 (95% CI, −0.0314 to 0.0273), whereas the mean improvement from M0 to M3 was 0.157 (95% CI, 0.103–0.217). Thus, V-LAD accounted for the largest gain in rank discrimination, whereas carotid plaque provided additional independent prognostic information and improved overall model fit without a consistent further increase in discrimination. Adding GLS to M3 did not improve model fit (likelihood ratio *p* = 0.846), and GLS was not independently associated with the primary endpoint (HR, 1.01; 95% CI, 0.90–1.14; *p* = 0.846).

As a complementary analysis, multivariable logistic regression was also performed using the same hierarchical model structure. These analyses were considered supportive and yielded a closely concordant hierarchy of model performance.

**Figure 2:**
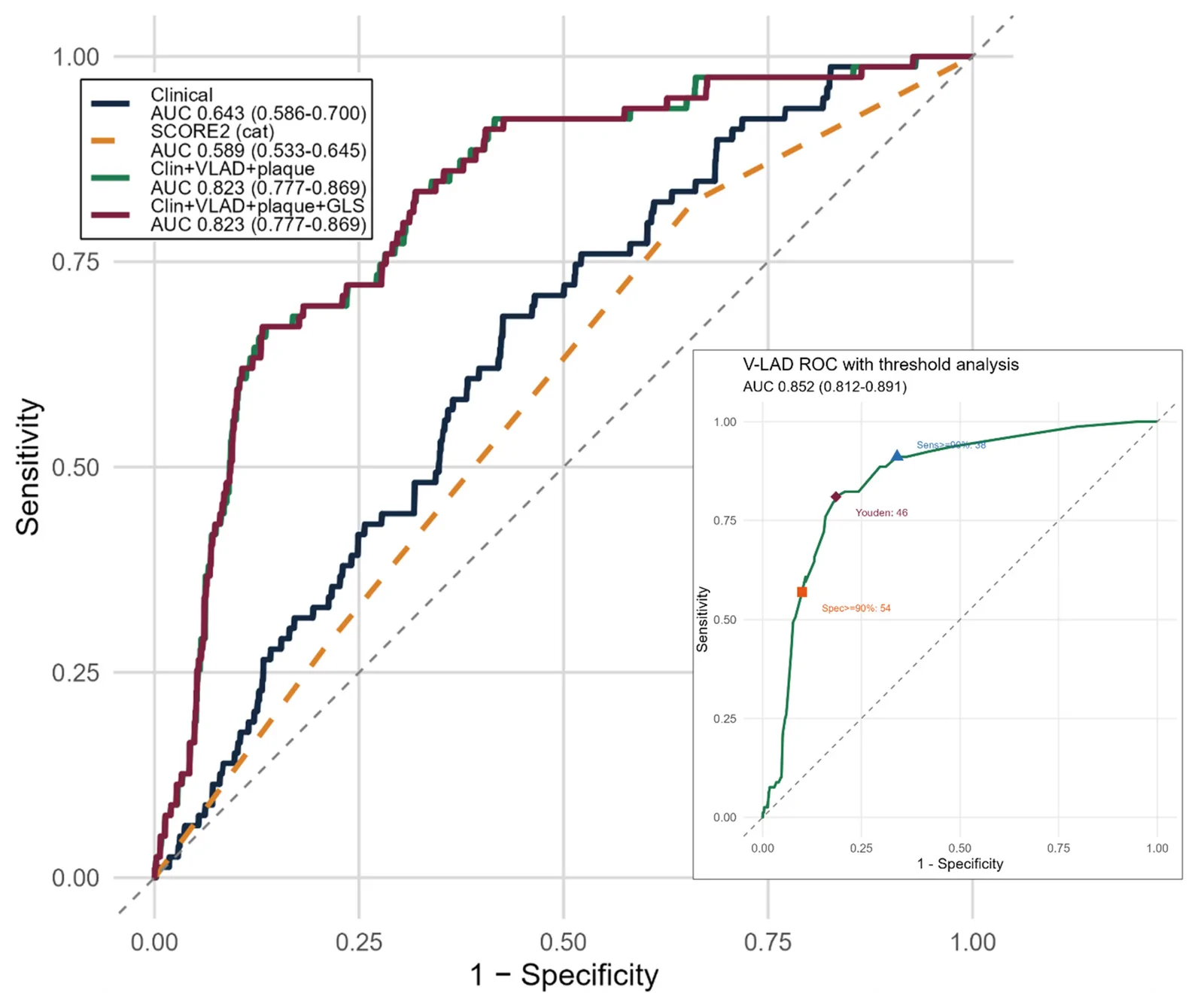
Logistic ROC curves for models with standalone V-LAD ROC analysis shown in the inset.

Adding continuous V-LAD to the clinical model (model M1 vs. M0) markedly improved discrimination, with the AUC increasing from 0.641 to 0.834 (and the Brier score decreasing from 0.0662 to 0.0642). Adding any carotid plaque alone to the clinical model (M2 vs. M0) also improved performance, although to a lesser extent, with an AUC of 0.738 and a Brier score of 0.0630. Figure 2 demonstrates that, among the ultrasound-derived variables, V-LAD provides the greatest incremental improvement in discriminative performance beyond the internal clinical model. The main combined imaging model (M3), integrating the clinical model with continuous V-LAD and any carotid plaque, showed substantially better discrimination than the clinical model alone, with an AUC of 0.823 (95% CI, 0.777–0.869) and the lowest Brier score of 0.0619; on matched rows, the increase in AUC was 0.181, and model fit improved significantly (likelihood ratio *p* < 0.0001). In multivariable logistic analysis (see Supplementary Table S1 for detailed multivariable data), both V-LAD and carotid plaque remained independently associated with the primary endpoint. The OR was 1.03 per cm/s increase in V-LAD (95% CI, 1.02–1.05; *p* < 0.001) and 3.77 for any carotid plaque (95% CI, 2.07–6.89; *p* < 0.001).

In the corresponding targeted logistic comparison restricted to matched rows, adding carotid plaque beyond the model containing clinical variables and V-LAD (model M3 vs. M1) improved model fit significantly (likelihood ratio *p* = 1.14 × 10^−5^) and reduced the Brier score from 0.0642 to 0.0619, although the AUC was slightly lower (0.834 vs. 0.823). By contrast, the addition of GLS to M3 (M4) did not improve discrimination or model fit and had an AUC of 0.823 (95% CI, 0.777–0.869), with a Brier score of 0.0624, a negligible change in AUC versus M3 on matched rows (−0.0001) and no significant improvement in fit (likelihood ratio *p* = 0.884). In the extended model, GLS was not independently associated with the primary endpoint (OR, 1.01; 95% CI, 0.89–1.15; *p* = 0.884), whereas both V-LAD and carotid plaque remained independently associated.

Apparent calibration did not show major deviations. Bootstrap internal validation of the supportive logistic models showed limited optimism for the main imaging model, with an apparent AUC of 0.823 and an optimism-corrected AUC of 0.810; the optimism-corrected Brier score was 0.0638 and the mean bootstrap test calibration slope was 0.927. Full internal validation metrics for all logistic models are reported in Supplementary Table S2.

### 3.5. Comparison with SCORE2

SCORE2 categories were analysed in their ordinal categorical form as a contextual comparator, acknowledging differences in endpoint definition and prediction horizon. In the endpoint-stratified baseline analysis, SCORE2-related risk domains were directionally less separated than the main imaging variables, particularly continuous V-LAD and carotid plaque burden.

In the complementary logistic analysis, ROC comparison (Figure 2) showed the rank order of performance across SCORE2, the clinical model, and the imaging models, with AUC values of 0.587 for SCORE2, 0.641 for the clinical model (M0), and 0.823 for the combined imaging model (M3). The ROC curve for V-LAD as a standalone variable (AUC 0.852; best Youden index cutoff, 46 cm/s) is also displayed Figure 2. In a sensitivity analysis restricted to non-diabetic participants, categorical SCORE2 showed a similar AUC of 0.597.

Supplementary Figure S3 graphically compares the discriminative performance of the models. Given the differences in endpoint definition and the shorter follow-up horizon in our study compared with those of the SCORE2 derivation cohort, this comparison should be interpreted as contextual only.

### 3.6. Reclassification Analyses

Complementary reclassification analyses derived from the logistic models are summarized in Supplementary Table S4 and displayed in Supplementary Figure S4. Compared with the internal clinical model (M0), the main imaging model (M3) yielded a very high continuous net reclassification improvement of 0.923 (95% CI, 0.670–1.144) and an integrated discrimination improvement of 0.087 (95% CI, 0.049–0.145).

The corresponding event and non-event components of the continuous net reclassification improvement were 0.468 (95% CI, 0.266–0.623) and 0.455 (95% CI, 0.369–0.561), respectively, indicating improvement in both upward reclassification among subjects with events and downward reclassification among those without events.

Adding global longitudinal strain on top of the combined V-LAD-plus-plaque model did not further improve reclassification. The addition of continuous V-LAD alone to the clinical model showed a continuous net reclassification improvement of 0.930 and an integrated discrimination improvement of 0.062, whereas the addition of carotid plaque alone yielded a continuous net reclassification improvement of 0.802 and an integrated discrimination improvement of 0.046. Global longitudinal strain alone provided a considerably smaller reclassification gain (continuous net reclassification improvement, 0.446; integrated discrimination improvement, 0.019).

Detailed reclassification matrices for events and non-events and heatmaps also for categorical V-LAD are reported in the Supplementary Materials (Supplementary Figure S5). Interestingly, the use of the model with categorical V-LAD plus any carotid plaque reclassified up or down more than half of the subjects, 649 subjects (59.2%), compared with the internal clinical model (M0).

Given the known sensitivity of continuous NRI to relatively small changes in predicted probabilities, these reclassification results are considered supportive rather than definitive evidence of clinical utility.

Decision curve analysis was also performed as a complementary analysis (Supplementary Figure S8). Imaging-enriched models generally provided greater net benefit than the clinical reference model over several clinically plausible thresholds; however, M3 did not uniformly outperform M1, further supporting a cautious interpretation of the incremental contribution of carotid plaque beyond V-LAD.

### 3.7. Sensitivity Analysis Excluding Angina

In a prespecified sensitivity analysis excluding angina, the number of events was limited (*n* = 12). In the Cox analysis, the main imaging model remained directionally supportive, with higher discrimination than the clinical model alone (Harrell C-index 0.906 vs. 0.801 on matched rows; Δ C-index 0.105; likelihood ratio χ^2^ = 18.01; *p* < 0.001). V-LAD remained independently associated with the endpoint (HR 1.48 per 10 cm/s; 95% CI 1.24–1.76; *p* < 0.001), whereas carotid plaque was not (HR 1.92; 95% CI 0.42–8.73; *p* = 0.398). Consistently, the complementary logistic analysis also showed higher discrimination with the main imaging model than with the clinical model alone (AUC 0.839 vs. 0.777; Δ AUC 0.062; likelihood ratio *p* = 0.0025). Given the very small number of events, this analysis should be regarded as exploratory and does not constitute validation of the findings for hard cardiovascular outcomes.

In an additional sensitivity analysis restricted to the coronary components of the primary endpoint (new-onset angina or acute coronary syndrome; *n* = 73), the incremental contribution of carotid plaque beyond V-LAD remained significant (likelihood ratio *p* < 0.001), indicating that the main findings were not driven by the atrial fibrillation or heart failure components of the composite endpoint. The coronary-specific analysis confirmed a strong contribution from plaque beyond V-LAD.

### 3.8. Secondary Threshold-Based Analysis

Secondary threshold analyses (fully reported and commented in the Supplementary Materials) examined binary and three-category parameterizations of V-LAD and GLS.

**Figure 3:**
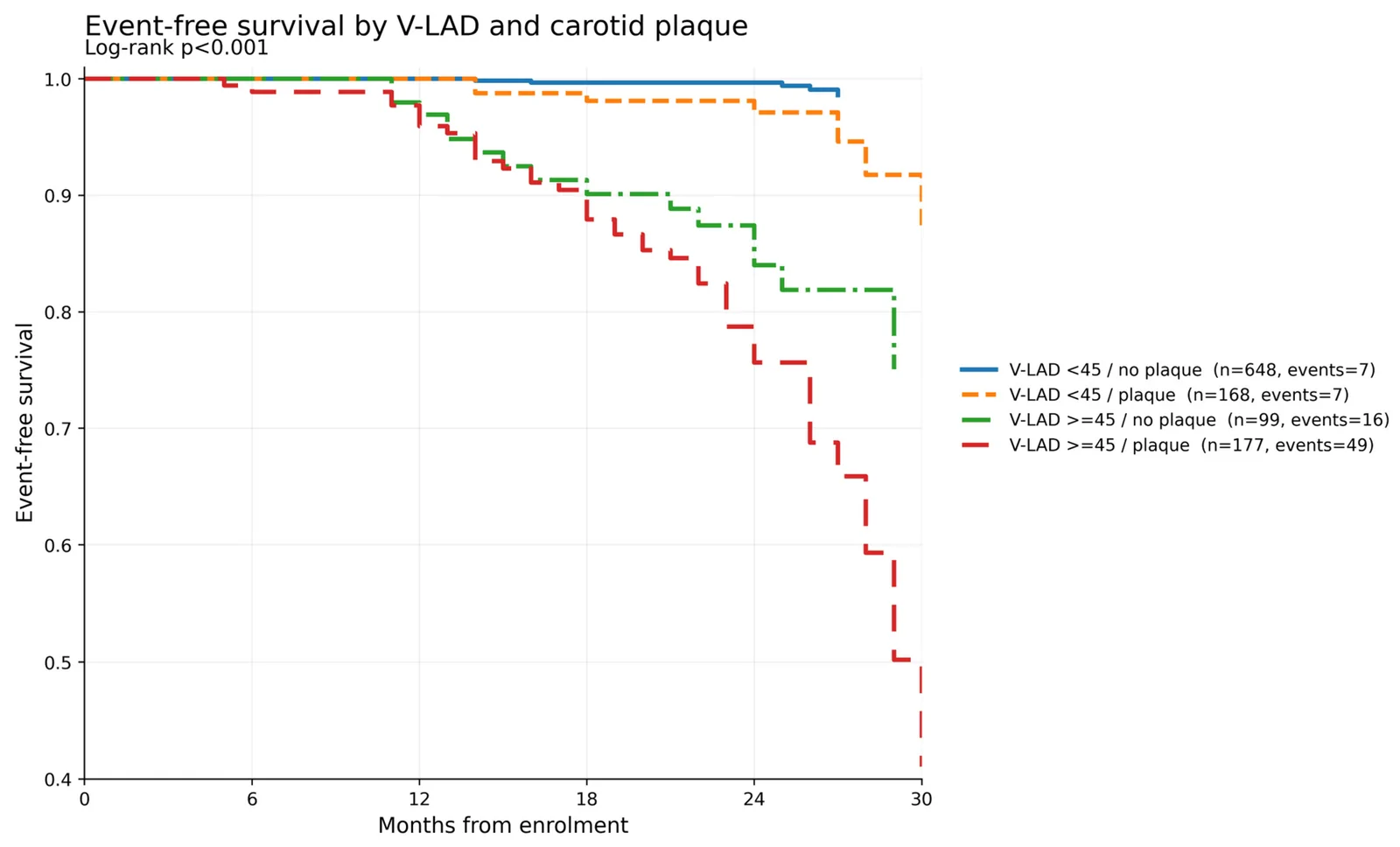
Kaplan–Meier curves for event-free survival according to V-LAD and carotid plaque status. Participants were stratified into four groups according to V-LAD < 45 or ≥45 cm/s and the absence or presence of carotid plaque. Differences among groups were significant by log-rank test (*p* < 0.001).

**Figure 4:**
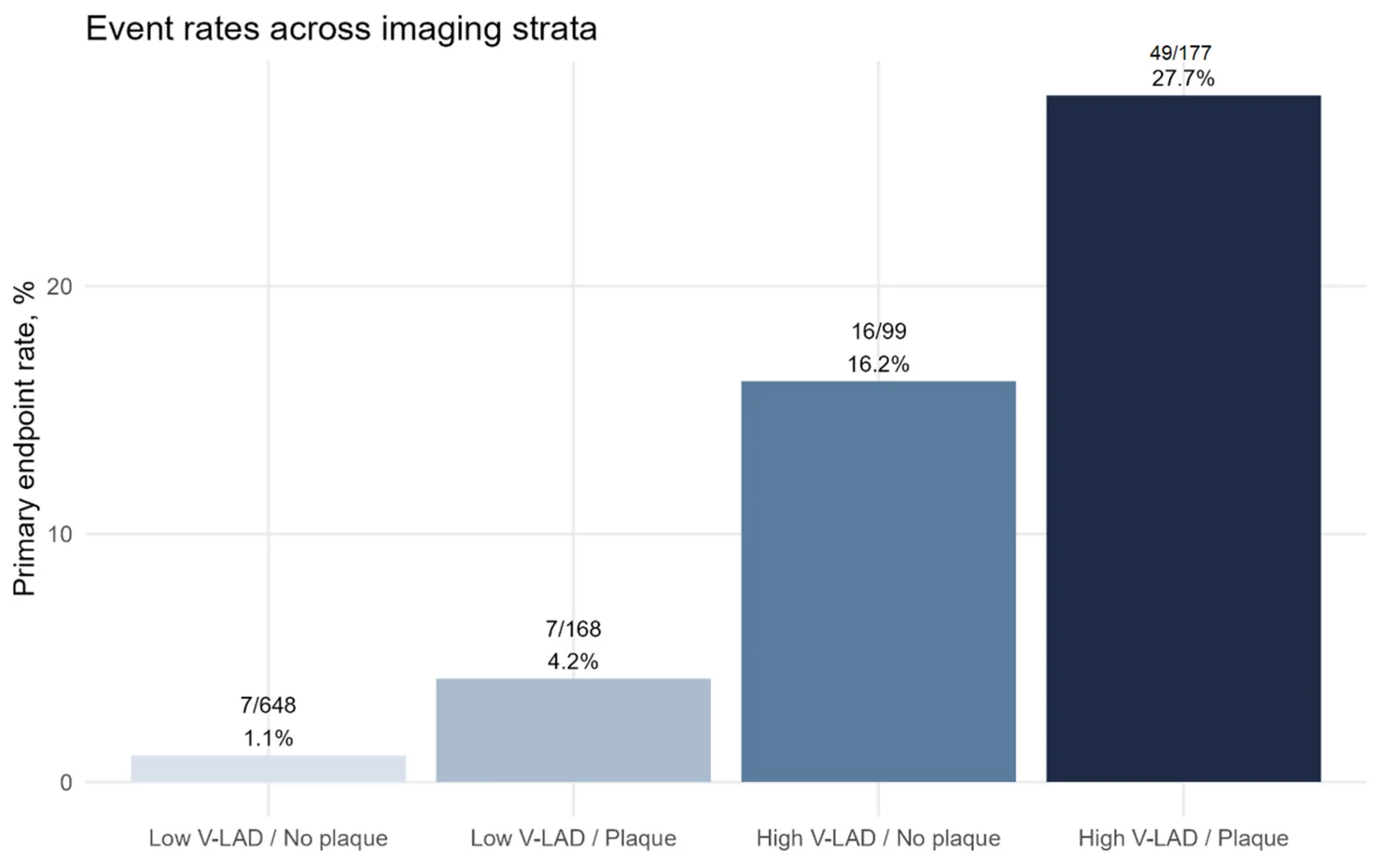
Primary endpoint rates across binary V-LAD and carotid plaque strata.

Kaplan–Meier analysis of the four strata defined by dichotomized V-LAD and carotid plaque status showed a marked separation in event-free survival over follow-up (log-rank *p* < 0.001; Figure 3). Figure 4 shows the clear stepwise pattern observed in the simplified binary imaging stratification combining dichotomized V-LAD and carotid plaque. Primary endpoint rates were 1.1% in subjects with low V-LAD and no plaque, 4.2% in those with low V-LAD and plaque, 16.2% in those with high V-LAD and no plaque, and 27.7% in those with both high V-LAD and plaque, consistent with a stepwise gradient in observed endpoint rates across the simplified binary imaging strata. Because these thresholds were empirically derived in the present cohort, this classification should be interpreted as exploratory and hypothesis-generating rather than as a validated clinical decision rule.

## 4. Discussion

The principal finding of this study is that the addition of two readily obtainable ultrasound markers, resting mid-left anterior descending (LAD) coronary flow velocity and the presence of at least one carotid plaque, substantially enhances prognostic stratification for a short-term composite cardiac endpoint in asymptomatic adults undergoing cardiovascular screening. In the primary time-to-event analysis, V-LAD provided the largest incremental gain in discrimination over the internal clinical reference model, whereas carotid plaque provided additional independent prognostic information and significantly improved overall model fit without a further increase in C-index beyond V-LAD alone. Complementary logistic analyses yielded a consistent hierarchy of model performance, with the combined imaging approach showing a marked improvement in discrimination versus the clinical model (AUC 0.823 vs. 0.641), accompanied by a robust gain in reclassification (continuous NRI 0.923; IDI 0.087). In contrast, the inclusion of global longitudinal strain (GLS) did not confer additional incremental value, indicating that the primary prognostic benefit in this setting is largely captured by the integration of coronary Doppler flow velocity and carotid plaque assessment.

A further notable observation is that this improvement was achieved using resting ultrasound parameters that are straightforward to obtain within a comprehensive cardiovascular examination, without the need for ionizing radiation, contrast agents, or costly equipment. This approach is particularly appealing in screening settings, where the clinical utility of a test depends not only on predictive performance but also on feasibility, reproducibility, safety, and seamless integration into routine preventive care pathways.

To date, V-LAD measurement is a niche technique, largely confined to specialized centres or experienced operators. However, it is not intrinsically complex and, with appropriate training and standardization of the technique, could be more widely adopted within the broader echocardiography community. The high technical feasibility observed in the present cohort supports its applicability in experienced hands, although reproducibility was not specifically reassessed within this study. The rationale for integrating multiple domains of cardiovascular risk assessment is both biologically and clinically compelling, as conventional risk factors, vascular imaging, and cardiac functional markers are likely to capture complementary and only partially overlapping determinants of future cardiovascular events. A recent analysis pooling data from three landmark screening studies showed that the addition of biomarker and imaging information to baseline SCORE2 or PCE improved short-term risk prediction in apparently healthy adults, although echocardiographic markers such as GLS and V-LAD were not available in that study [14].

In another large screening study, clinical risk factors, biomarkers, and echocardiographic data including GLS were collected, but carotid ultrasound was not performed [7].

To the best of our knowledge, a combined carotid and cardiac ultrasound strategy incorporating carotid plaque, V-LAD, and GLS has previously been explored only in relation to angiographic coronary artery disease detection. The present study extends this approach to prospective prognostic stratification for clinical cardiac events [15,16]. In our cohort, SCORE2 was included solely as a contextual comparator, given differences in both endpoint definition and follow-up duration relative to its derivation setting. Within this exploratory contextual comparison, its discriminative performance was modest (AUC 0.587) and lower than that of the internal clinical and imaging models in the complementary logistic analysis. This should not be interpreted as a limitation of SCORE2 per se, but rather as reflecting that, in this specific screening population and for a short-term composite endpoint, direct measures of subclinical vascular and coronary abnormalities may provide additional prognostic information beyond conventional risk estimation.

Among imaging variables, V-LAD showed the strongest incremental contribution. In the primary Cox analysis, its addition increased the Harrell C-index from 0.653 to 0.824, and V-LAD remained independently associated with the primary endpoint. Complementary logistic analysis provided concordant findings, with an AUC of 0.834. Threshold analyses identified a cutoff of approximately 46 cm/s, with good sensitivity and specificity, and risk stratification demonstrated a clear gradient in event rates. These findings are biologically plausible, as increased resting LAD diastolic flow velocity likely reflects integrated coronary pathophysiology, including epicardial disease and microvascular dysfunction.

The presence of a carotid plaque, even when anatomically minimal and of limited apparent clinical significance, provided meaningful incremental prognostic information. Although its isolated contribution to discrimination was smaller than that of V-LAD, carotid plaque remained independently associated with the endpoint in the combined model. Moreover, its addition to a V-LAD–based model significantly improved overall model fit, despite the absence of a further increase in C-index; a similar pattern was observed in the complementary logistic analysis, in which the AUC was slightly lower for M3 than for M1. This pattern suggests that carotid plaque and coronary Doppler capture complementary dimensions of risk: the former reflecting systemic atherosclerotic burden, and the latter serving as a more specific functional marker of coronary physiology. Supportive reclassification heatmaps supported this interpretation, demonstrating a more appropriate redistribution of individuals with events toward higher-risk categories and those without events toward lower-risk categories following the inclusion of both parameters.

By contrast, GLS did not materially enhance model performance once V-LAD and carotid plaque were included. The lack of a meaningful increase in C-index, absence of improvement in model fit, and only minimal additional gain in supportive reclassification analyses all indicate that GLS contributed limited independent prognostic information in this context. This does not detract from the established role of GLS as a marker of myocardial function; rather, it suggests that, in an asymptomatic screening population with largely preserved cardiac structure and function, its value may be outweighed by markers more directly related to subclinical atherosclerosis and coronary functional impairment.

### Limitations

Several limitations should be acknowledged. First, follow-up was relatively short in this interim analysis (median 25 months), and the number of non-angina events was limited; accordingly, the findings primarily reflect short-term risk stratification. Although the sensitivity analysis excluding angina was directionally consistent with the main findings, it included only 12 events and should therefore be considered exploratory rather than evidence of validation for harder cardiovascular outcomes. Second, the composite endpoint differed from that used for SCORE2 derivation, both in definition and time horizon, making this comparison contextual. Third, this was a single-center study, and external validation in independent cohorts with different demographic and risk profiles is required before broader generalization. Because the angina component required downstream functional or anatomic testing, partial verification and referral bias cannot be excluded, particularly if screening findings influenced subsequent diagnostic pathways. In particular, elevated V-LAD contributed to selection for downstream CCTA according to the study diagnostic pathway, and knowledge of abnormal screening findings may also have influenced subsequent symptom perception or reporting. Although all adjudicated angina events represented new-onset symptoms after enrolment and required objective confirmation, these potential sources of ascertainment bias cannot be completely eliminated in an unblinded screening study. For this reason, coronary revascularization was excluded from the primary endpoint, and a sensitivity analysis excluding angina was performed. Fourth, transthoracic LAD Doppler is partly operator-dependent, and the high feasibility achieved in this cohort may not be reproducible in less experienced laboratories. Finally, atrial fibrillation was retained because it formed part of the prespecified broader cardiac composite endpoint; the coronary-specific sensitivity analysis was performed to assess whether the findings were driven by non-coronary components, yielding findings consistent with the primary analysis.

## 5. Conclusions

In a prospective cardiovascular screening cohort of asymptomatic adults, an ultrasound-based approach combining V-LAD and carotid plaque assessment significantly and consistently improved prognostic stratification beyond a parsimonious clinical reference model, whereas the incremental contribution of GLS appeared limited, if any. V-LAD provided the largest gain in discrimination, while carotid plaque added independent prognostic information and improved overall model fit without a further increase in discrimination beyond V-LAD alone. Complementary logistic analyses provided concordant findings, including improved discrimination and reclassification versus the clinical model. In contextual comparison, the primary imaging model showed higher discrimination than categorical SCORE2 for this short-term composite endpoint.

These findings highlight the potential value of integrating coronary Doppler velocity and carotid plaque assessment into ultrasound-based cardiovascular screening strategies and provide a strong rationale for external validation in larger, independent cohorts with longer follow-up and more conventional atherosclerotic cardiovascular disease endpoints.

## Figures and Tables

**Table 1:** Baseline Characteristics, Labs and Imaging Data.

Block	Variable	Overall (*n* = 1094)	No Endpoint (*n* = 1015)	Endpoint (*n* = 79)	*p* Value
Demographics	Age, years	60.00 (57, 65)	60.00 (56, 65)	62.00 (58.50, 67)	0.035
	Male sex	549 (50.2%)	499 (49.2%)	50 (63.3%)	0.0218
	BMI, kg/m^2^	24.90 (22.69, 27.77)	24.91 (22.69, 27.78)	24.24 (22.47, 27.64)	0.55
	Hypertension	385 (35.2%)	341 (33.6%)	44 (55.7%)	<0.001
	Hypercholesterolemia	622 (56.9%)	562 (55.4%)	60 (75.9%)	<0.001
	Diabetes mellitus	85 (7.8%)	77 (7.6%)	8 (10.1%)	0.561
	Smoking	494 (45.2%)	453 (44.6%)	41 (51.9%)	0.257
	Family history of CAD	585 (53.5%)	536 (52.9%)	49 (62.0%)	0.145
	Systolic BP, mmHg	132.00 (120, 140)	130.00 (120, 140)	132.00 (131, 145)	<0.001
	Waist circumference, cm	91 (82, 100)	91 (82.25, 100)	87 (78, 92)	0.00331
	Sedentary lifestyle	654 (59.9%)	606 (59.8%)	48 (61.5%)	0.859
Laboratory	Blood glucose	98.00 (92, 105)	98.00 (92.00, 104.00)	99.00 (93.50, 108.00)	0.116
	Creatinine	0.89 (0.79, 1)	0.89 (0.79, 1.00)	0.90 (0.80, 1.00)	0.871
	Uric acid	5.15 (4.30, 6)	5.20 (4.30, 6.00)	5.10 (4.50, 5.95)	0.957
	NT-proBNP	71.00 (45, 112)	70.00 (45.00, 110.00)	98.00 (55.00, 123.50)	0.0137
	Triglycerides	99.00 (77, 130)	98.00 (77, 129.5)	105.00 (78.50, 130)	0.686
	Total cholesterol	201.00 (180, 226.75)	200.00 (180, 225)	210.00 (187, 233)	0.102
	LDL cholesterol	129.50 (107, 151)	129.00 (107, 150)	142.00 (112.50, 160)	0.0802
	Calculated LDL	119.00 (100, 140)	118.00 (100, 140)	129.00 (100.50, 147)	0.0478
	HDL cholesterol	59.00 (50.00, 69.00)	59.00 (50.00, 69.00)	61.00 (48.00, 67.00)	0.662
	TSH	2.00 (1.47, 2.73)	2.00 (1.45, 2.74)	1.97 (1.58, 2.43)	0.951
	Troponin I	3.30 (2.50, 6)	3.30 (2.50, 6)	4 (3, 9.75)	0.0214
	SCORE2, %	6.30 (3.90, 9.70)	6.20 (3.82, 9.67)	7.90 (5.77, 12.72)	0.0031
Carotid Ultrasound	Any carotid plaque	345 (31.5%)	289 (28.5%)	56 (70.9%)	<0.001
	Carotid IMT thickness, mm	1.20 (0.90, 1.30)	1.11 (0.80, 1.30)	1.20 (1.20, 1.30)	0.00145
Echocardiography	LVEF, %	60 (58, 62)	60 (58, 62)	59 (58, 60)	<0.001
	GLS, %	−21.00 (−22, −19)	−21.00 (−22, −19)	−19.00 (−21, −18)	<0.001
	LV end-diastolic diameter, mm	48 (45, 50)	48 (45, 50)	49 (46, 52)	0.00248
	LV segmental WM abnormality	14 (1.3%)	13 (1.3%)	1 (1.3%)	1
	Left atrial diameter, mm	38 (36, 39)	37 (36, 39)	38 (36, 40)	<0.001
	Left atrial volume, mL	27 (24, 29)	27 (24, 29)	28 (26, 30)	<0.001
	E/e′ ratio	9 (8, 10)	9 (7, 10)	10 (9, 11)	<0.001
	V-LAD, cm/s	35.00 (33.00, 45.00)	34.00 (33.00, 40.00)	56.00 (49.00, 63.50)	<0.001
Follow-up	Follow-up, months	25.00 (22.00, 27.00)	25.00 (22.00, 27.00)	21.00 (14.00, 26.00)	<0.001

Data are presented as median [IQR] for continuous variables and n (%) for categorical variables. Abbreviations: BMI, body mass index; BP, blood pressure; CAD, coronary artery disease; EF, ejection fraction; GLS, global longitudinal strain; HDL, high-density lipoprotein; IMT, intima-media thickness; IQR, interquartile range; LAD, left anterior descending artery; LDL, low-density lipoprotein; LV, left ventricle; NT-proBNP, N-terminal pro-brain natriuretic peptide; SCORE2, Systematic Coronary Risk Estimation 2; TSH, thyroid-stimulating hormone. *p*-values in red indicate statistical significance (*p* < 0.05); Mann–Whitney U test for continuous variables, chi-squared test for categorical.

**Table 2:** Primary Cox model performance.

Model	Description	N	Events	Harrell C-Index *	Δ C-Index vs. M0	Incremental Likelihood Ratio Comparison	*p* Value
M0	Clinical reference	1093	79	0.653	0.000	Reference	—
M1	Clinical + V-LAD	1091	79	0.824	0.171	vs. M0: χ^2^ 57.22 (1 df)	<0.001
M2	Clinical + carotid plaque	1093	79	0.730	0.077	vs. M0: χ^2^ 46.77 (1 df)	<0.001
M3	Clinical + V-LAD + carotid plaque	1091	79	0.816	0.163	vs. M1: χ^2^ 25.82 (1 df)	<0.001
M4	Clinical + V-LAD + carotid plaque + GLS	1080	79	0.814	0.160	vs. M3: χ^2^ 0.04 (1 df)	0.846

* Harrell C-indices are discrimination summaries from the conventional Cox models. Final likelihood ratio tests derive from the final inferential Cox models including the same systolic blood pressure × log(time/24) interaction in M0–M4; V-LAD, carotid plaque, and GLS were modelled with constant hazard ratios. M0 included age, male sex, smoking, systolic blood pressure, and total cholesterol. For completeness, M3 vs. M0: LR χ^2^ 83.03 (2 df), *p* < 0.001; M4 vs. M0: LR χ^2^ 82.64 (3 df), *p* < 0.001. The significant M1-to-M3 likelihood ratio test indicates improved model fit from carotid plaque beyond V-LAD, despite no further increase in C-index.

## Data Availability

Data will be provided in anonymous format for research upon reasonable request.

## Supplementary Materials

The following supporting information can be downloaded at: https://www.mdpi.com/article/10.3390/diagnostics16182903/s1, Supplementary Figure S1: Study flowchart; Supplementary Figure S2: Distribution of V-LAD, GLS, and carotid plaque status according to primary endpoint status; Supplementary Figure S3: Model discrimination summary comparing Harrell C-index from the primary Cox models with AUC from the logistic models; Supplementary Figure S4: Logistic reclassification summary; Supplementary Figure S5: Logistic reclassification matrices and heatmaps; Supplementary Figure S6: Observed primary endpoint rates across V-LAD risk strata; Supplementary Figure S7: Observed primary endpoint rates across GLS risk strata; Supplementary Figure S8: Decision-curve analysis with bootstrap-corrected net benefit; Supplementary Table S1: Multivariable logistic regression coefficients; Supplementary Table S2: Logistic internal validation; Supplementary Table S3: Logistic model performance; Supplementary Table S4: Logistic reclassification analyses; Supplementary Table S5: Selected bootstrap-corrected decision-curve net benefit.
