# Supplementary material for: Resting Ultrasound Coronary Flow Velocity and Carotid Plaque Improve Clinical Risk Stratification in Asymptomatic Adults Undergoing Cardiovascular Screening
Source: Diagnostics (Basel). 2026 Sep 9;16(18):2903. doi: 10.3390/diagnostics16182903 (PMC13605245; doi:10.3390/diagnostics16182903)

Supplementary Tables

Supplementary Table S1. multivariable logistic regression coefficients.

Model	Term	OR	95% CI lower	95% CI upper	P value
M0	Age	1.026	0.984	1.070	0.229
M0	Male sex	1.658	1.019	2.699	0.042
M0	Smoking	1.316	0.827	2.096	0.247
M0	Systolic blood pressure	1.016	1.001	1.031	0.034
M0	Total cholesterol	1.005	0.999	1.012	0.127
M3	Age	0.980	0.937	1.026	0.390
M3	Male sex	0.959	0.569	1.618	0.877
M3	Smoking	1.282	0.781	2.106	0.326
M3	Systolic blood pressure	1.007	0.990	1.024	0.411
M3	Total cholesterol	0.997	0.990	1.004	0.400
M3	V-LAD, per 1 cm/s	1.035	1.021	1.049	<0.001
M3	Any carotid plaque	3.773	2.066	6.890	<0.001
M4	Age	0.980	0.936	1.026	0.396
M4	Male sex	0.943	0.553	1.610	0.830
M4	Smoking	1.279	0.779	2.102	0.331
M4	Systolic blood pressure	1.007	0.990	1.024	0.409
M4	Total cholesterol	0.997	0.990	1.004	0.347
M4	V-LAD, per 1 cm/s	1.034	1.019	1.050	<0.001
M4	Any carotid plaque	3.775	2.054	6.937	<0.001

Model	Term	OR	95% CI lower	95% CI upper	P value
M4	GLS, per 1 percentage point	1.009	0.890	1.145	0.884

Intercepts are omitted for clarity. M0 included age, male sex, smoking, systolic blood pressure, and total cholesterol; M3 added V-LAD and any carotid plaque; M4 added GLS to M3. OR, odds ratio; CI, confidence interval; GLS, global longitudinal strain; V-LAD, resting left anterior descending coronary artery peak diastolic flow velocity. These logistic models are supportive to the primary Cox time-to-event analysis.

Supplementary Table S2. logistic internal validation.

Model	Description	N	Events	Apparent AUC	Mean AUC optimism	Optimism-corrected AUC	Apparent Brier	Optimism-corrected Brier	Calibration slope
M0	Clinical	1093	79	0.641	0.031	0.610	0.0662	0.0664	0.809
M1	Clinical + V-LAD	1091	79	0.834	0.017	0.817	0.0642	0.0663	0.898
M2	Clinical + carotid plaque	1093	79	0.738	0.021	0.717	0.0630	0.0640	0.933
M3	Clinical + V-LAD + carotid plaque	1091	79	0.823	0.012	0.810	0.0619	0.0638	0.927
M4	Clinical + V-LAD + carotid plaque + GLS	1080	79	0.823	0.018	0.805	0.0624	0.0644	0.909

Internal validation used 500 bootstrap resamples. Calibration slope refers to the mean bootstrap test calibration slope. AUC, area under the receiver-operating characteristic curve; Brier, Brier score. These results are supportive to the primary Cox analysis.

Supplementary Table S3. Logistic model performance.

Model	Description	N	Events	AUC (95% CI)	Brier	LR test vs M0	Delta AUC vs M0
M0	Clinical	1093	79	0.641 (0.584-0.698)	0.0662	Reference	0.000
M1	Clinical + V-LAD	1091	79	0.834 (0.790-0.877)	0.0642	<0.001	0.192
M2	Clinical + carotid plaque	1093	79	0.738 (0.682-0.794)	0.0630	<0.001	0.096
M3	Clinical + V-LAD + carotid plaque	1091	79	0.823 (0.777-0.869)	0.0619	<0.001	0.181
M4	Clinical + V-LAD + carotid plaque + GLS	1080	79	0.823 (0.777-0.869)	0.0624	<0.001	0.182
SCORE2	Categorical contextual comparator	1094	79	0.587 (0.531-0.643)	0.0667	-	-

Likelihood-ratio comparisons are from the logistic models. SCORE2 is retained only as a contextual comparator because its endpoint definition and prediction horizon differ from those of the present study. The primary model-performance analysis is the Cox analysis reported in the main manuscript.

Supplementary Table S4. logistic reclassification analyses.

Comparison	Metric	Estimate	95% CI lower	95% CI upper	N	Events
M0 vs M3	Total NRI	0.923	0.670	1.144	1091	79
M0 vs M3	NRI in events	0.468	0.266	0.623	1091	79
M0 vs M3	NRI in non-events	0.455	0.369	0.561	1091	79
M0 vs M3	IDI	0.087	0.049	0.145	1091	79
M0 vs M4	Total NRI	0.919	0.690	1.167	1080	79
M0 vs M4	NRI in events	0.468	0.301	0.646	1080	79
M0 vs M4	NRI in non-events	0.451	0.365	0.561	1080	79
M0 vs M4	IDI	0.087	0.052	0.161	1080	79

Continuous NRI and IDI are reported as complementary metrics and should be interpreted cautiously because continuous NRI can be sensitive to small changes in predicted probabilities. NRI, net reclassification improvement; IDI, integrated discrimination improvement.

Supplementary Table S5. Selected bootstrap-corrected decision-curve net benefit.

Risk threshold	M0	M1	M3	Treat all	Treat none
0.05	0.0276	0.0442	0.0432	0.0236	0.0000
0.07	0.0140	0.0401	0.0326	0.0026	0.0000
0.10	0.0007	0.0304	0.0290	-0.0307	0.0000
0.15	-0.0038	0.0032	0.0180	-0.0913	0.0000
0.20	-0.0006	-0.0035	0.0036	-0.1595	0.0000

Decision-curve analysis was supportive. Imaging-enriched models provided greater net benefit than the clinical reference at several thresholds, but M3 did not uniformly dominate M1. Values shown are selected thresholds from the bootstrap-corrected analysis.

Supplementary Figures

Supplementary Figure S1. Study flowchart.

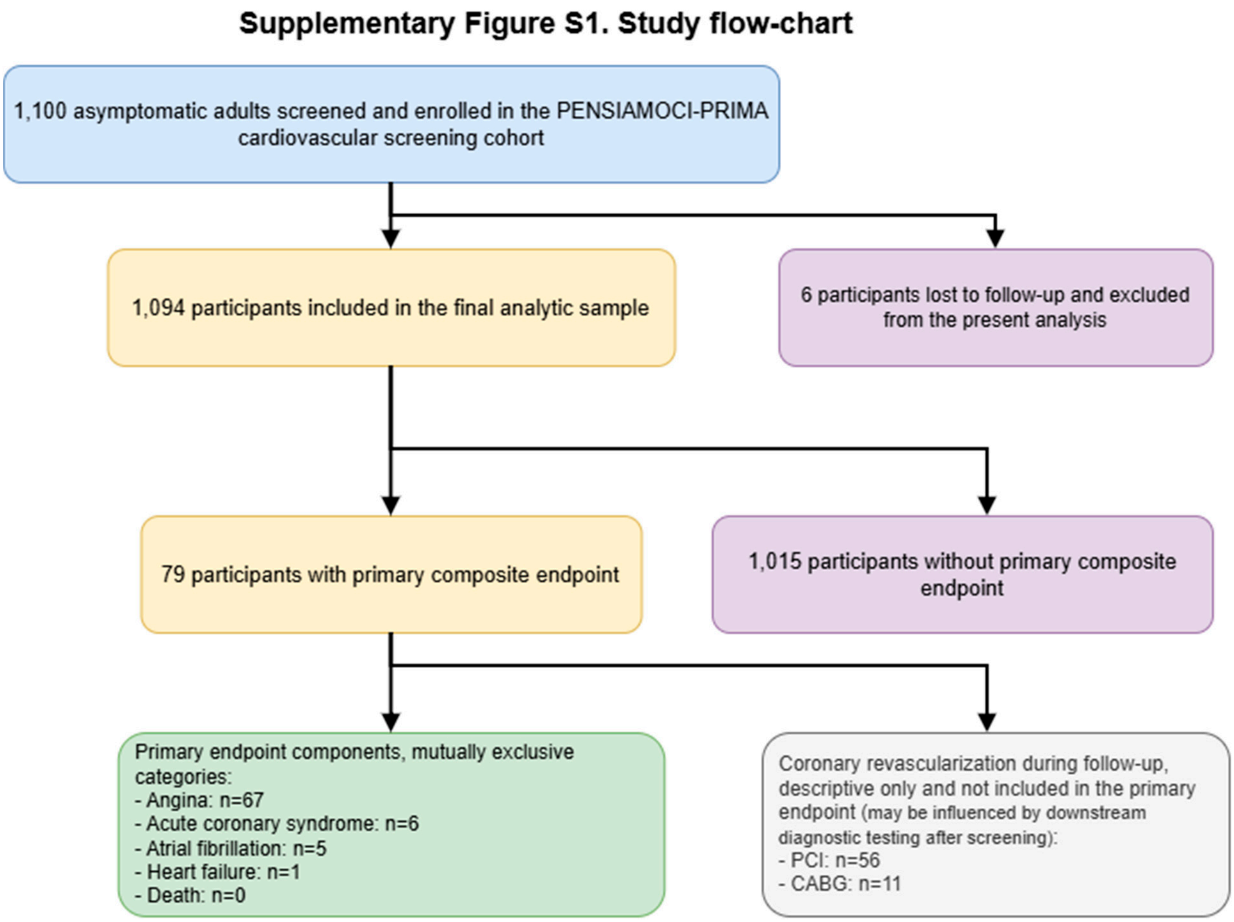

Supplementary Figure S2. Distribution of V-LAD, GLS, and carotid plaque status according to primary endpoint status.

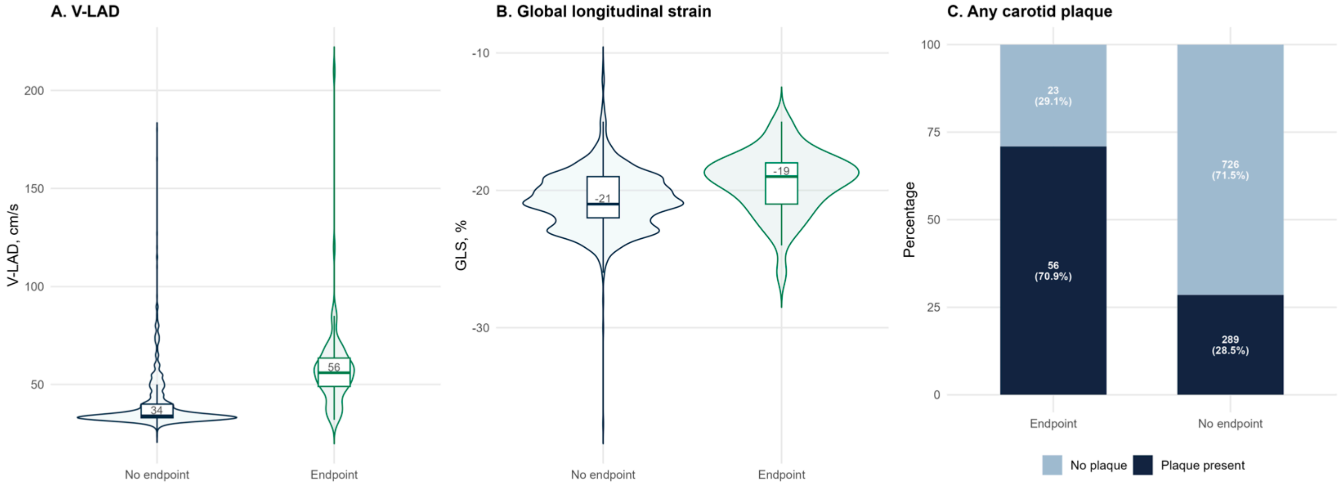

.

Supplementary Figure S3. Model discrimination summary comparing Harrell C-index from the primary Cox models with AUC from the logistic models.

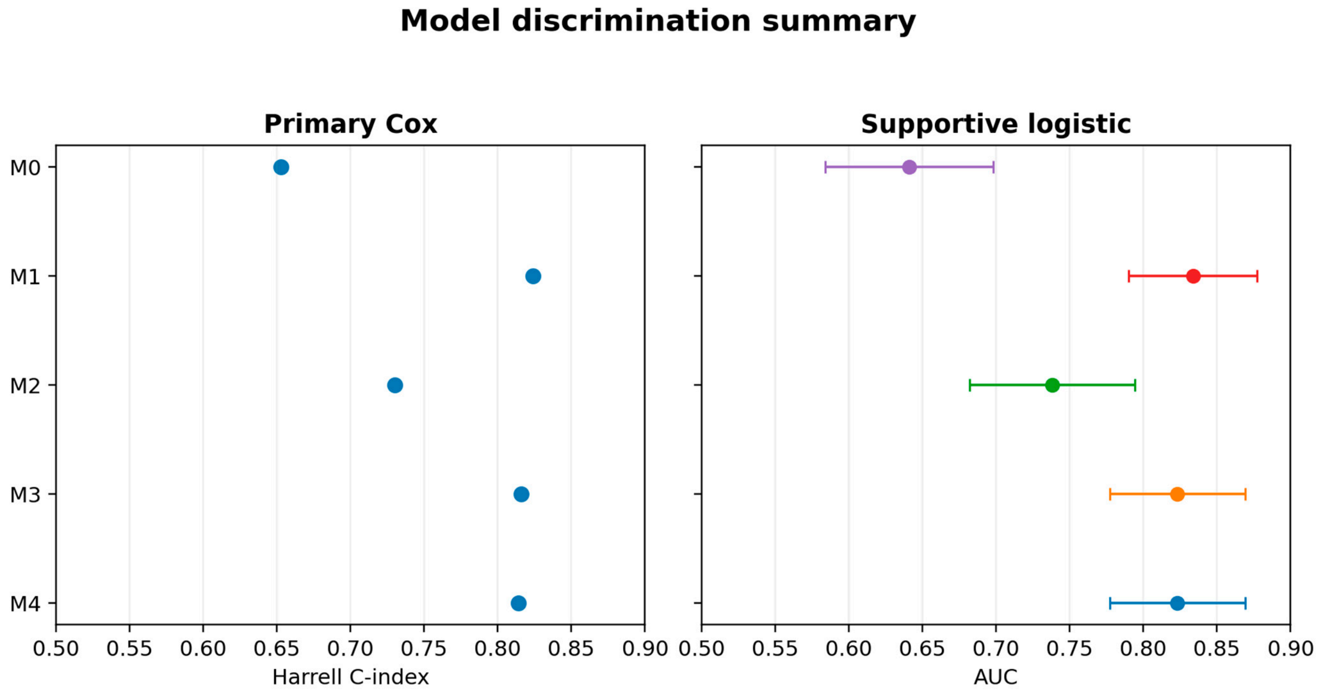

95% CIs displayed for logistic AUC only. Cox C-indices are shown as discrimination summaries.

Supplementary Figure S4. logistic reclassification summary.

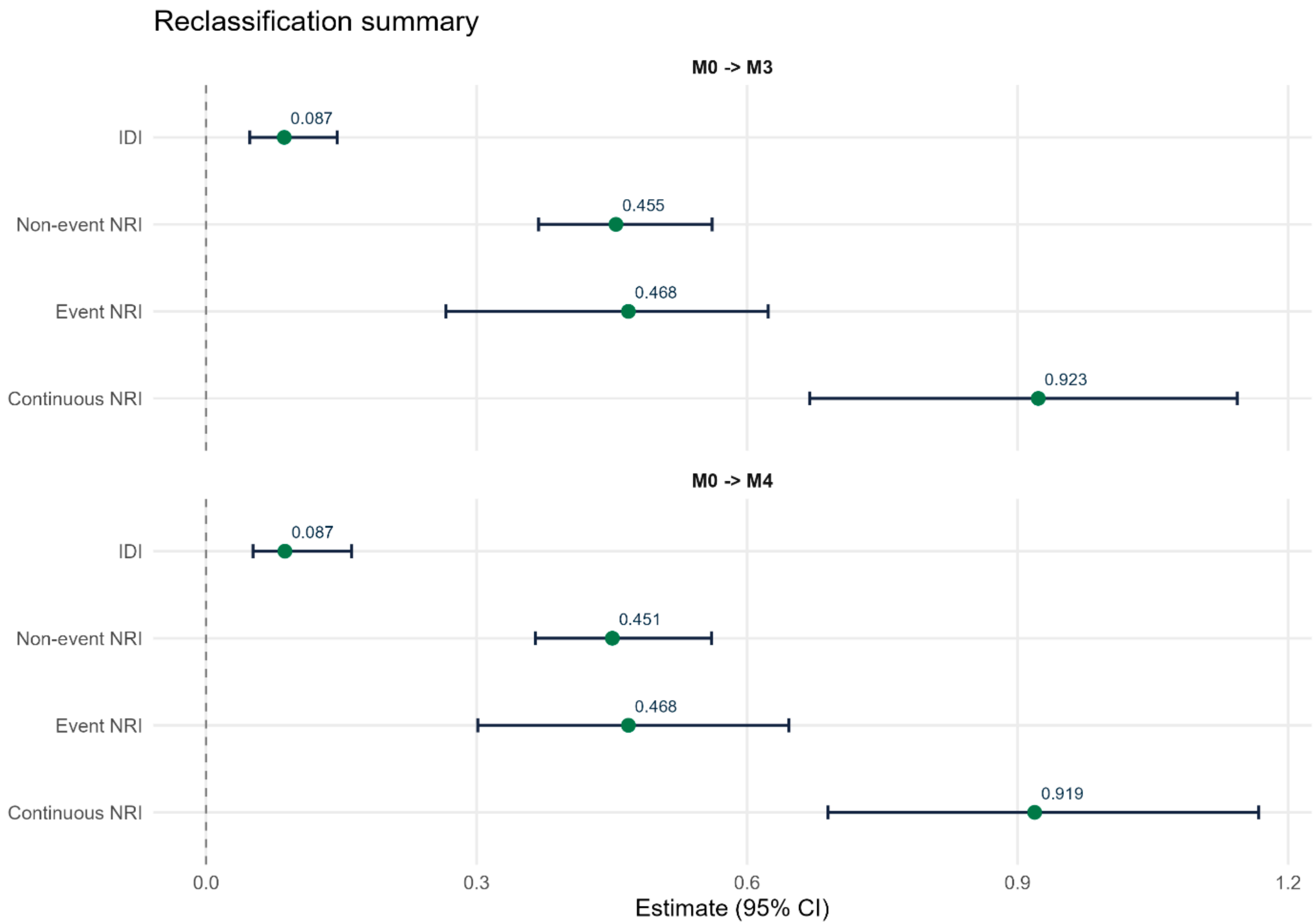

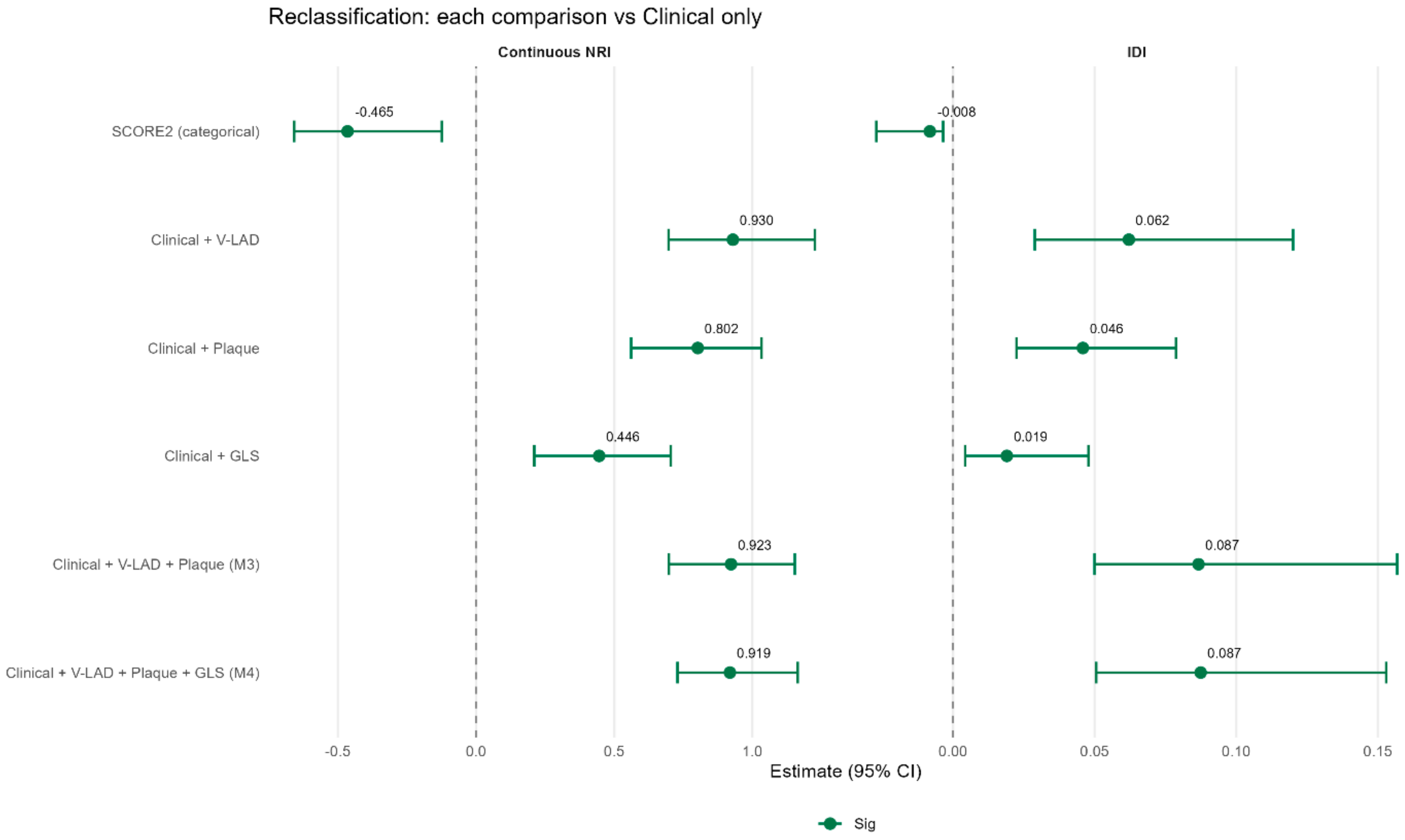

Reclassification: each comparison vs Clinical only
Continuous NRI
IDI
SCORE2 (categorical)
Clinical + V-LAD
Clinical + Plaque
Clinical + GLS
Clinical + V-LAD + Plaque (M3)
Clinical + V-LAD + Plaque + GLS (M4)
-0.465
0.930
0.802
0.446
0.923
0.919
-0.008
0.062
0.046
0.019
0.087
0.087
-0.5
0.0
0.5
1.0
0.00
0.05
0.10
0.15
Estimate (95% CI)
Sig

Supplementary Figure S5. logistic reclassification matrices and heatmaps.

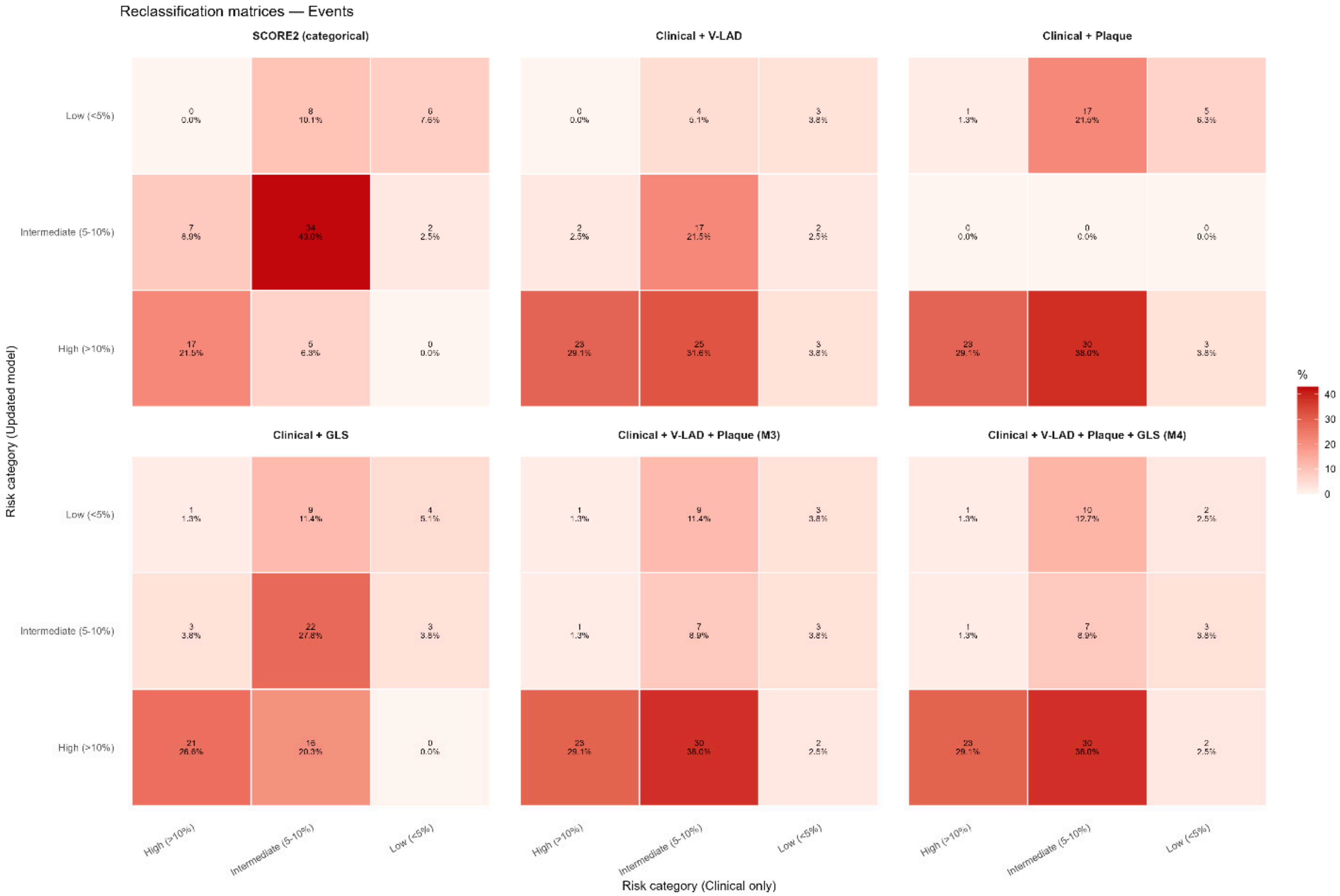

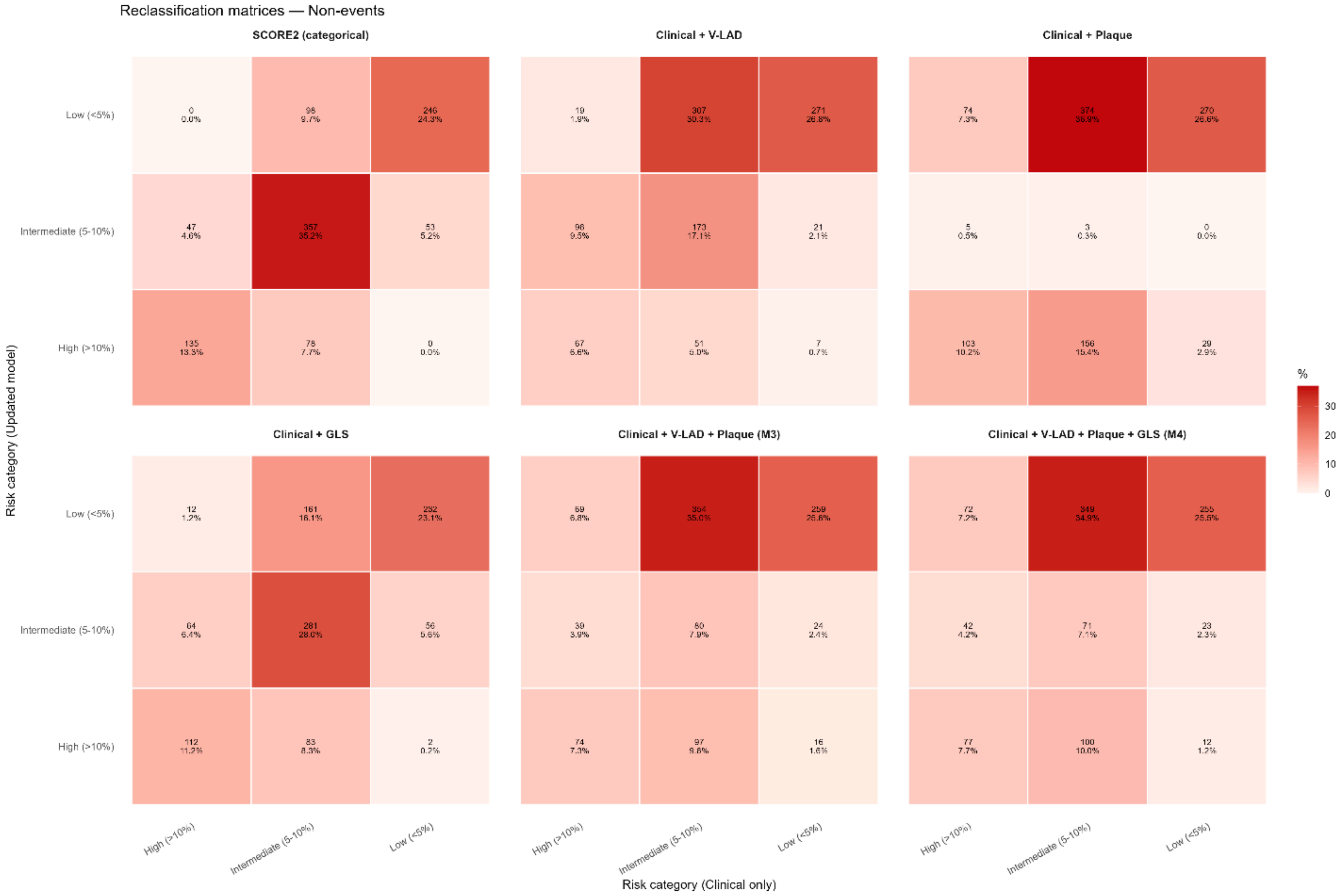

Reclassification matrices — Non-events
SCORE2 (categorical)
Clinical + V-LAD
Clinical + Plaque
Clinical + GLS
Clinical + V-LAD + Plaque (M3)
Clinical + V-LAD + Plaque + GLS (M4)
Risk category (Updated model)
Low (<5%)
Intermediate (5-10%)
High (>10%)
0 0.0%
98 9.7%
246 24.3%
47 4.6%
357 35.2%
53 5.2%
135 13.3%
78 7.7%
0 0.0%
19 1.9%
307 30.3%
271 26.8%
96 9.5%
173 17.1%
21 2.1%
67 6.6%
51 5.0%
7 0.7%
74 7.3%
374 36.9%
270 26.6%
5 0.5%
3 0.3%
0 0.0%
103 10.2%
156 15.4%
29 2.9%
12 1.2%
161 16.1%
232 23.1%
64 6.4%
281 28.0%
56 5.6%
112 11.2%
83 8.3%
2 0.2%
69 6.8%
354 35.0%
259 25.6%
39 3.9%
80 7.9%
24 2.4%
74 7.3%
97 9.6%
16 1.6%
72 7.2%
349 34.9%
255 25.5%
42 4.2%
71 7.1%
23 2.3%
77 7.7%
100 10.0%
12 1.2%
High (>10%)
Intermediate (5-10%)
Low (<5%)
Risk category (Clinical only)
%
30
20
10
0

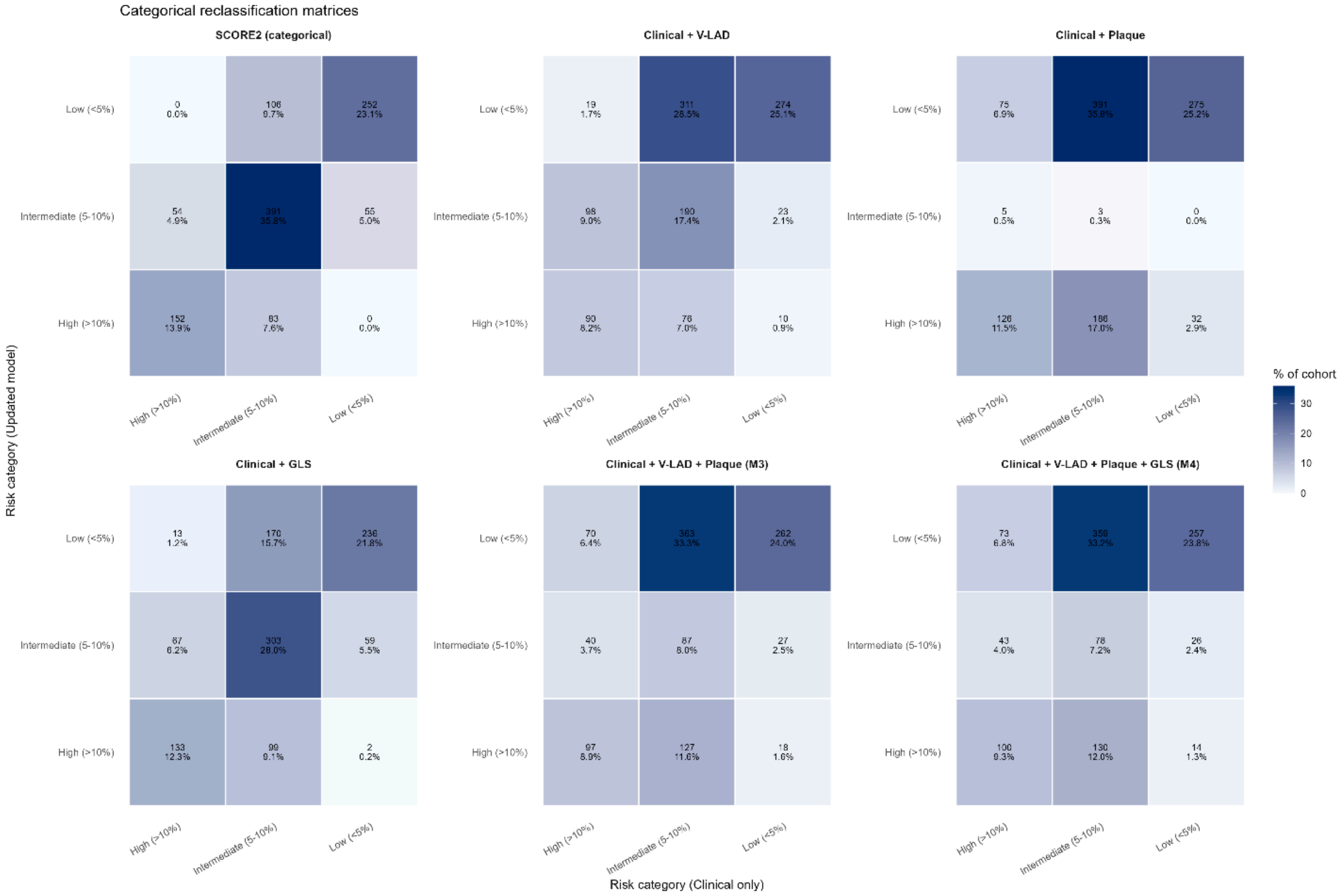

Categorical reclassification matrices
SCORE2 (categorical)
Clinical + V-LAD
Clinical + Plaque
Clinical + GLS
Clinical + V-LAD + Plaque (M3)
Clinical + V-LAD + Plaque + GLS (M4)
Low (<5%)
Intermediate (5-10%)
High (>10%)
Risk category (Updated model)
Risk category (Clinical only)
% of cohort
30
20
10
0

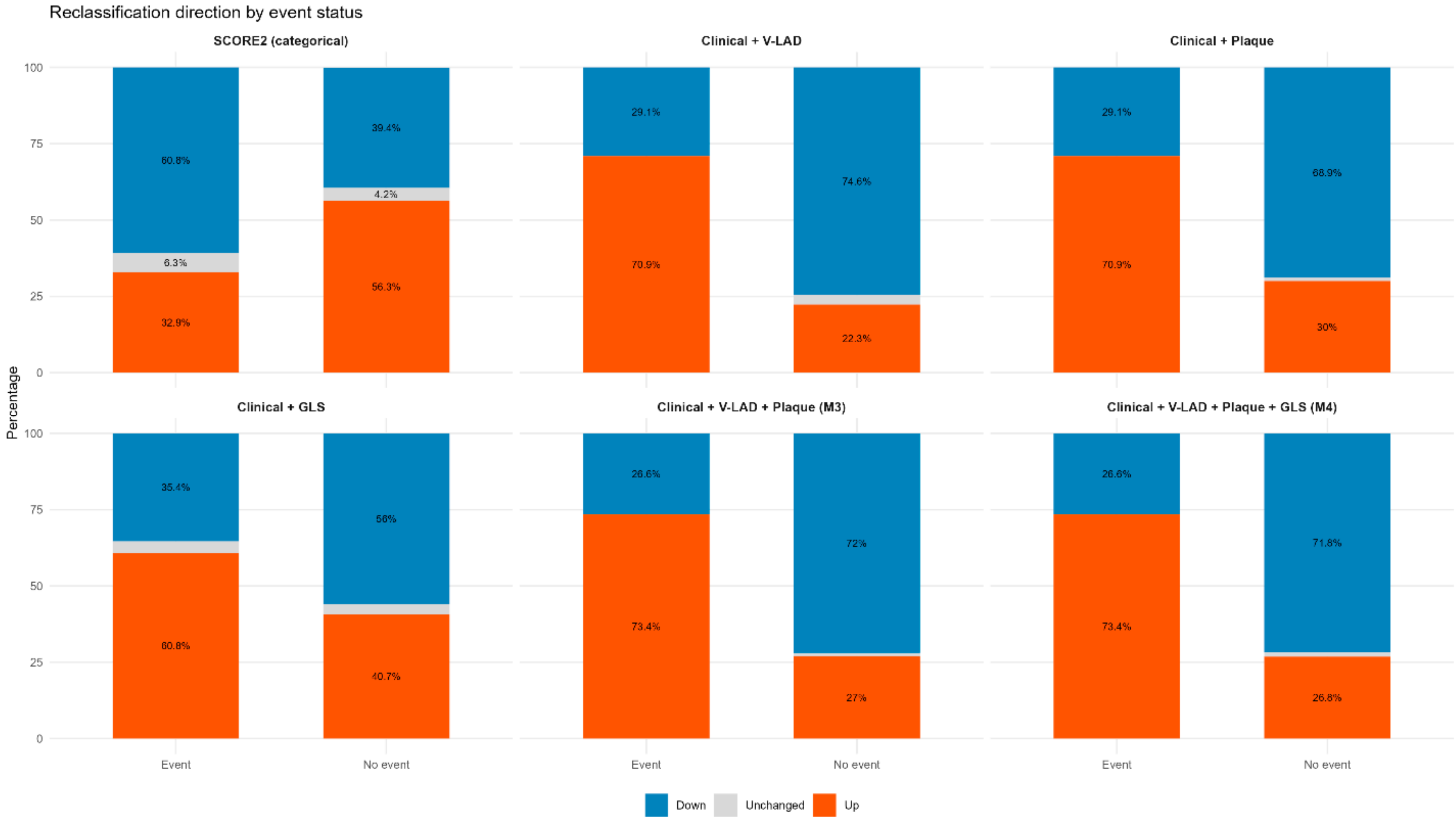

Interpretation of reclassification analyses. The heatmaps provide a visual complement to the summary NRI and IDI estimates. Directionally, the addition of V-LAD and carotid plaque redistributed a greater proportion of participants with events toward higher predicted risk and a proportion of participants without events toward lower predicted risk. However, because these are logistic analyses and continuous NRI is sensitive to relatively small changes in predicted probabilities, the heatmaps and reclassification indices should not be interpreted as definitive evidence of clinical utility.

Supplementary Figure S6. Observed primary endpoint rates across V-LAD risk strata.

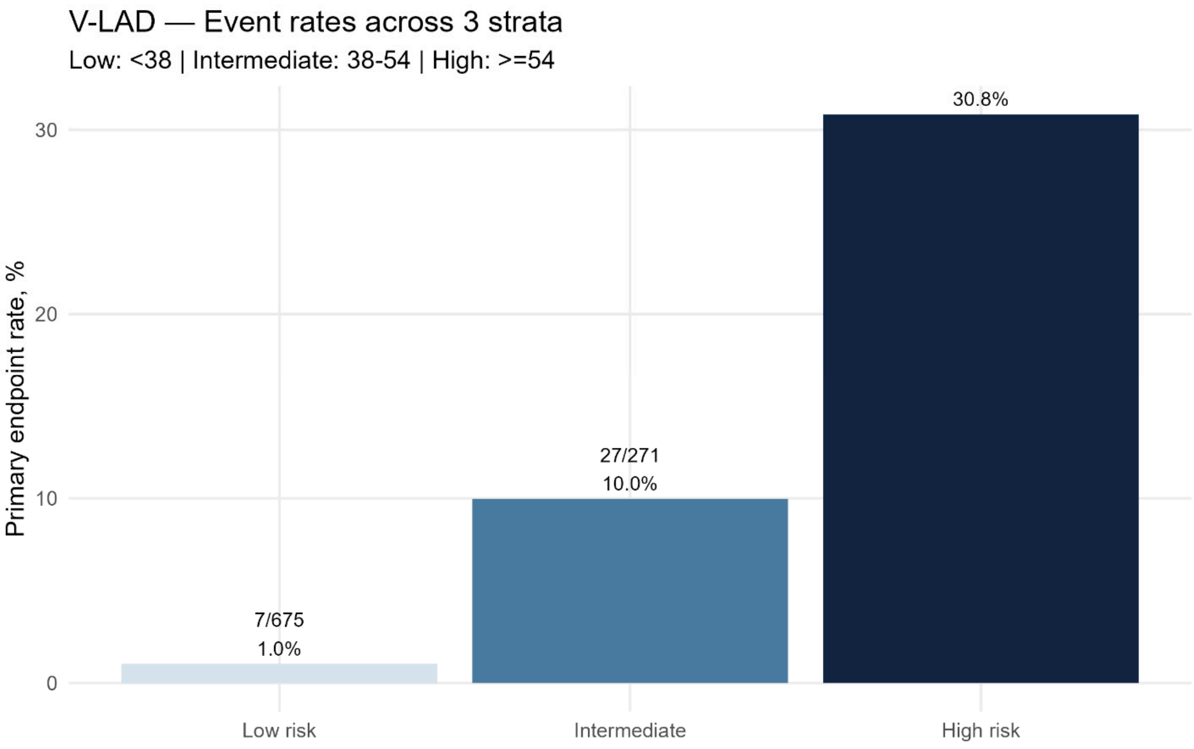

For GLS, threshold-based parameterizations did not improve discrimination compared with continuous modelling. The AUC was 0.709 for continuous GLS, 0.652 for the binary cutoff, and 0.650 for the three-category classification. A descriptive gradient in event rates was nevertheless observed across GLS strata, with rates of 4.3%, 7.1%, and 21.4% in the low-, intermediate-, and high-risk groups, respectively.

Supplementary Figure S7. Observed primary endpoint rates across GLS risk strata.

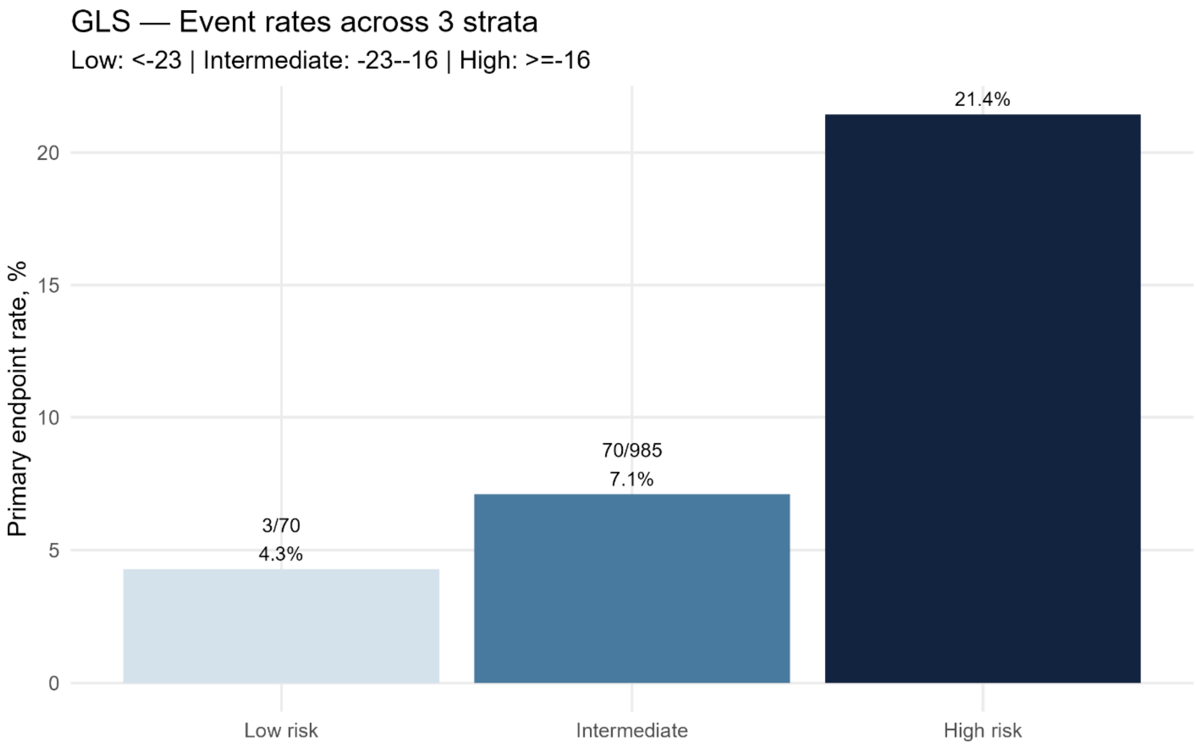

Supplementary Figure S8. decision-curve analysis with bootstrap-corrected net benefit.

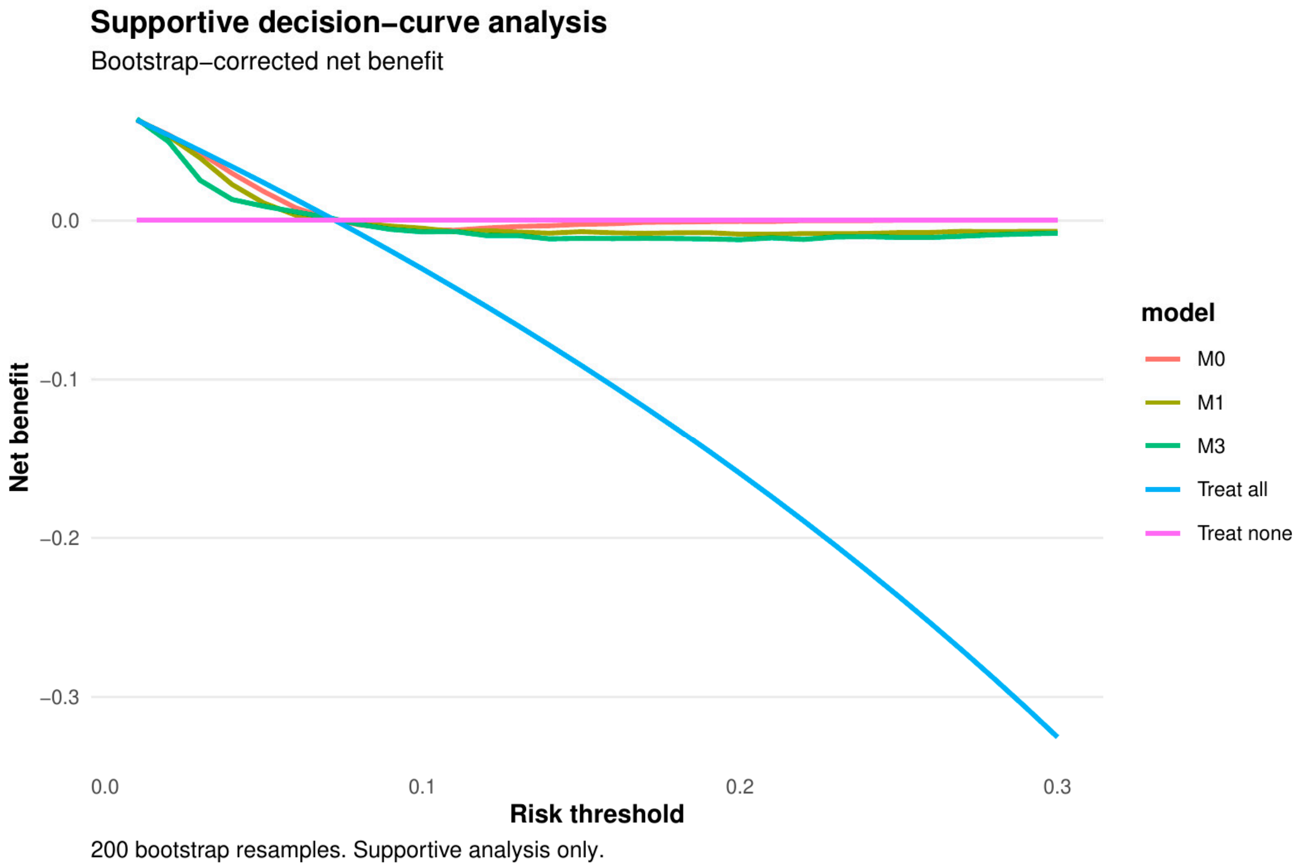

Supplement: Supplementary file 1 [file diagnostics-16-02903-s001.zip › diagnostics-4529985-supplementary.pdf]
